# Mechanistic Insights into the Formation of Thermoelectric TiNiSn from In Situ Neutron Powder Diffraction

**DOI:** 10.1021/acs.chemmater.3c00393

**Published:** 2023-04-26

**Authors:** Sonia A. Barczak, Blair F. Kennedy, Ivan da Silva, Jan-Willem G. Bos

**Affiliations:** † Institute of Chemical Sciences and Centre for Advanced Energy Storage and Recovery, School of Engineering and Physical Sciences, Heriot-Watt University, Edinburgh EH14 4AS, U.K.; ‡ ISIS Facility, Rutherford Appleton Laboratory, Harwell Oxford, Didcot OX11 0QX, U.K.

## Abstract

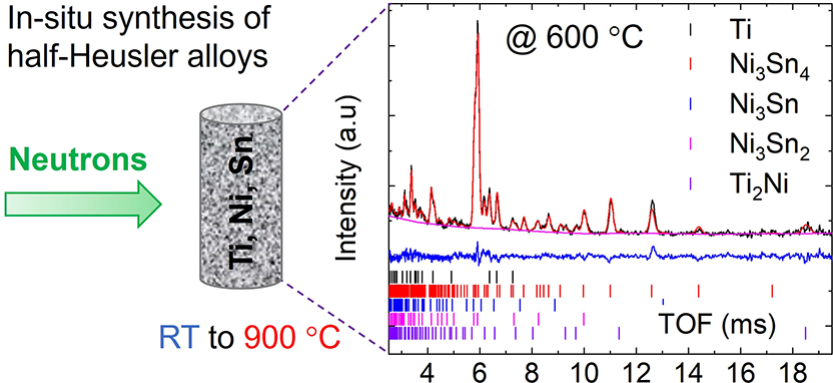

Half-Heusler alloys are leading contenders for application in thermoelectric generators. However, reproducible synthesis of these materials remains challenging. Here, we have used in situ neutron powder diffraction to monitor the synthesis of TiNiSn from elemental powders, including the impact of intentional excess Ni. This reveals a complex sequence of reactions with an important role for molten phases. The first reaction occurs upon melting of Sn (232 °C), when Ni_3_Sn_4_, Ni_3_Sn_2_, and Ni_3_Sn phases form upon heating. Ti remains inert with formation of Ti_2_Ni and small amounts of half-Heusler TiNi_1+y_Sn only occurring near 600 °C, followed by the emergence of TiNi and full-Heusler TiNi_2*y*’_Sn phases. Heusler phase formation is greatly accelerated by a second melting event near 750–800 °C. During annealing at 900 °C, full-Heusler TiNi_2*y*’_Sn reacts with TiNi and molten Ti_2_Sn_3_ and Sn to form half-Heusler TiNi_1+*y*_Sn on a timescale of 3–5 h. Increasing the nominal Ni excess leads to increased concentrations of Ni interstitials in the half-Heusler phase and an increased fraction of full-Heusler. The final amount of interstitial Ni is controlled by defect chemistry thermodynamics. In contrast to melt processing, no crystalline Ti–Sn binaries are observed, confirming that the powder route proceeds via a different pathway. This work provides important new fundamental insights in the complex formation mechanism of TiNiSn that can be used for future targeted synthetic design. Analysis of the impact of interstitial Ni on the thermoelectric transport data is also presented.

## 1 Introduction

The past 2 decades have seen rapid advances in thermoelectric performance, suggesting that commercial adaptation of thermoelectric power generation is within reach.^1−13^ However, widespread application is hindered by high costs compared to other sources of (renewable) electricity. A major challenge for thermoelectrics is to translate high performance into cheaper materials that can be readily processed and incorporated in thermoelectric generators at scale.^14,15^

Half-Heusler (HH) alloys are key candidates for thermoelectric application due to a combination of good performance, favorable engineering properties, and low cost.^16−18^ The current best materials are n-type XNiSn (X = Ti, Zr, Hf), p-type XCoSb, and p-type X’FeSb (X’ = Nb, Ta).^19−22^ Recently, complex compositions have also attracted attention (e.g., vacancy X’_0.8_CoSb, double and high-entropy HHs).^23−26^ For all HH materials, careful processing is vital to extract the best performance with significant variations between different studies.^16,17,27−29^ The performance of a thermoelectric material is quantified by a figure of merit, *zT* = (*S*^2^σ/κ)*T*.^30^ Here, *S* is the Seebeck coefficient (the voltage response to a temperature gradient), σ is the electrical conductivity, κ is the sum of the lattice (κ_lat_) and electronic (κ_e_) thermal conductivity, and *T* is the absolute temperature. The overall generator efficiency is proportional to the temperature difference that is being used and the average *zT* of the p- and n-type materials.^31^

n-type XNiSn HH alloys have been reported with *zT* ≤ 1.3–4 at 500 °C.^32−37^ Most of the high-*zT* compositions use substantial quantities of expensive Hf to maximize alloy disorder. An alternative route to reduce κ_lat_ is the use of interstitial metals,^38−43^ which embed substantial disorder in the HH structure and can lead to good overall performance. For example, *zT* = 0.8 at 500 °C for compositions with interstitial Cu, which suppresses κ_lat_ but does not degrade the overall electronic performance.^44,45^ By contrast, interstitial Ni degrades the electronic mobility and, despite the favorable impact on κ_lat_, limits the possible improvements of *zT*.^46−48^ Controlling the amount of interstitial Ni is therefore very important for preparing high-performance XNiSn HHs, but there is no direct insight into how it is incorporated in the HH structure during synthesis.

**Figure 1:**
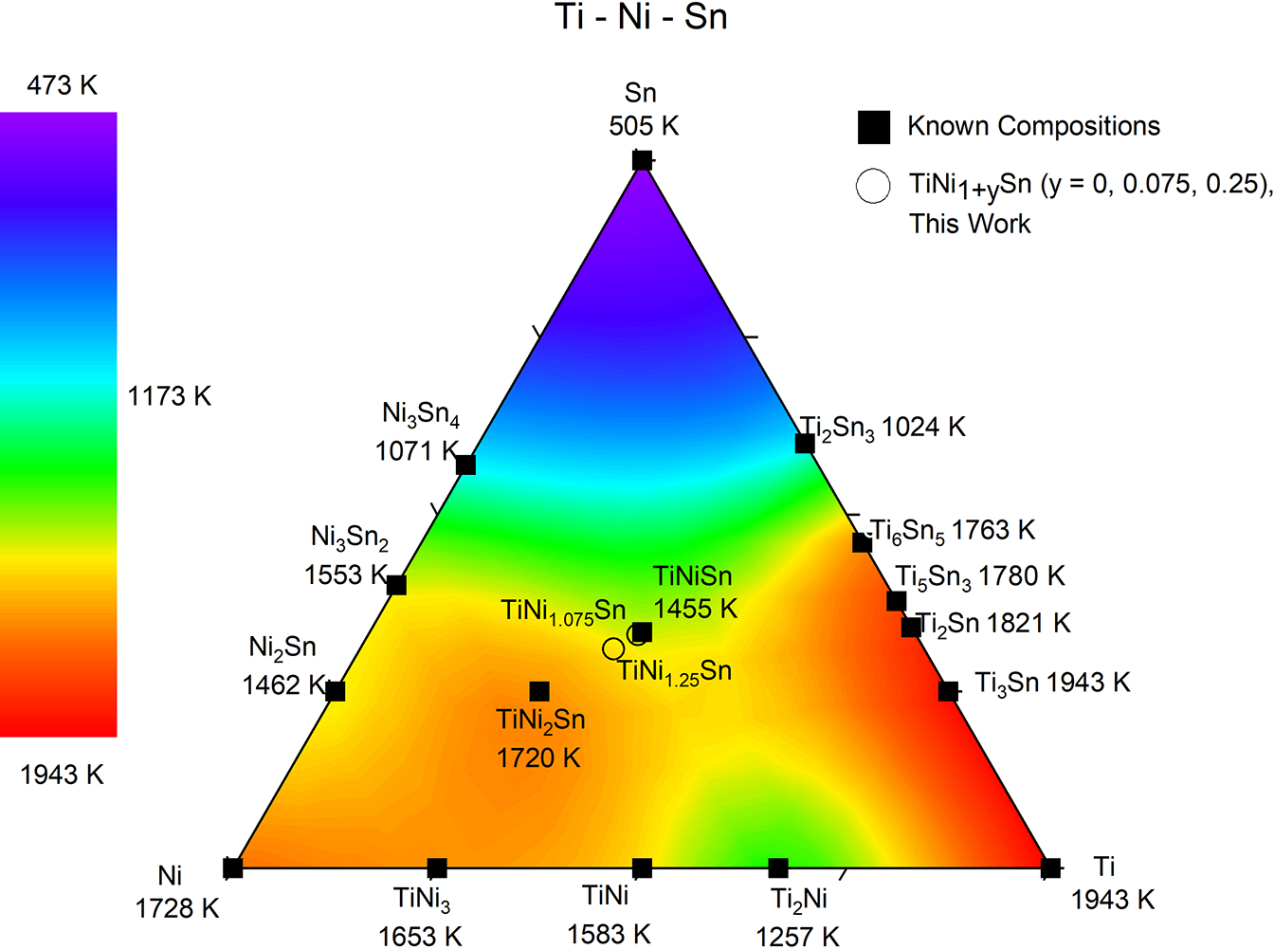
Interpolated contour map of the ternary Ti–Ni–Sn system. Included are known phases and their melting temperatures. TiNiSn, TiNi_1.075_Sn, and TiNi_1.25_Sn are the compositions produced and analyzed in this study. Redrawn using data from Douglas et al. Phase stability and property evolution of biphasic Ti–Ni–Sn alloys for use in thermoelectric applications. *Journal of Applied Physics***2014**, *115*, 043720. Copyright 2014, AIP publishing.^51^

The common route to prepare XNiSn HH alloys is via melting, and this has been widely investigated using (ex situ) experimental and theoretical phase diagram studies.^32−36^ ZrNiSn and HfNiSn melt congruently and are relatively straightforward to prepare^49^ but still suffer from incorporation of interstitials.^42^ Formation of TiNiSn follows an indirect pathway. From ex situ studies, it is known that samples quenched from the melt contain full-Heusler (FH) TiNi_2*y*’_Sn, high-melting point (MP) Ti–Sn binaries, and elemental Sn.^50^ These phases then gradually react to form the HH phase at typical annealing temperatures of 800–1000 °C. This pathway can be understood from the Ti–Ni–Sn phase diagram shown in Figure 1, which shows a false color plot of MPs.^51^ In essence, the reaction follows phase stability with the higher-MP compositions TiNi_2_Sn (MP = 1447 °C) and Ti–Sn binaries (Ti_6_Sn_5_ and Ti_5_Sn_3_ are common. MP = 1477–1507 °C) forming first on cooling from the melt. At lower temperatures (below the incongruent MP of TiNiSn at 1182 °C), these phases (and Sn) react to form TiNiSn, which is often found to contain substantial amounts of Ni interstitials (i.e., TiNi_1+*y*_Sn composition). An outstanding question is if these interstitials are controlled by thermodynamics (the stability of Ni interstitial defects in the HH phase) or are kinetically trapped Ni during conversion of TiNi_2_Sn into TiNiSn.

A less explored route is direct reaction between elemental powders,^38,39,44,45,52^ including via self-propagating combustion synthesis.^53,54^ This is advantageous from a scaling perspective as it avoids melting and is a less energy-intense and greener route to produce HH materials. Post-synthesis diffraction and microscopy often reveal the presence of FH TiNi_2*y*’_Sn and elemental Sn, suggesting that these again play an important role in HH formation.

Here, we present the first direct study of the formation of TiNi_1+*x*_Sn (*x* = 0, 0.075, and 0.25) from elemental powders. In this article, *x* is used to denote nominal composition, while *y* and *y*’ are used to indicate experimental HH (TiNi_1+*y*_Sn) and FH (TiNi_2*y*’_Sn) compositions obtained from Rietveld analysis. In situ neutron powder diffraction (NPD) reveals substantial differences compared to high-temperature melt-based routes. This includes the initial formation of Ni–Sn phases and the importance of molten phases for rapid phase transformations with full product formation occurring within hours. Similar to the melt-based routes, HH formation also involves the FH phase, so neither route avoids this phase in the reaction pathway. Analysis of the temperature and time dependence of the Ni interstitial defect concentration reveals that this is controlled by thermodynamics, with Ni only “freezing-in” below 500–600 °C. We also present an analysis of the impact of interstitial Ni on the thermoelectric properties, which consolidates literature knowledge. This study provides important new insights that will aid the design of improved scalable processing strategies.

## 2 Experimental Section

### 2.1 In Situ NPD Study

Powder mixtures with nominal TiNi_1+*x*_Sn (*x* = 0, 0.075, and 0.25) composition were prepared from high-purity elemental precursors (Alfa Aesar; Ti, 325 mesh; Ni, 120 mesh; Sn, 100 mesh; ≥99.8% purity). The powders were thoroughly mixed using an agate mortar and pestle and cold-pressed into 13 mm diameter × 2 mm height disks using 10 ton uniaxial pressure. The pellets were quartered using a scalpel and stacked to a height of ∼40 mm (corresponding to the beam size and ∼8 gram of the sample) and then tightly wrapped in V foil and placed in a standard 10 mm diameter V can. NPD data was collected using the General Materials Diffractometer (GEM) instrument at the ISIS facility, Rutherford Appleton Laboratory, UK. All samples were heated in a high-vacuum furnace at temperatures up to 900 °C with a ramp rate of 3 °C/min. Here, 900 °C was chosen to mimic laboratory synthesis conditions, where samples are commonly synthesized at this temperature in vacuum-sealed silica tubes. Diffraction patterns were collected for 10 min throughout the experiment; hence, each pattern corresponds to a 30 °C temperature interval during the ramping stage. At 900 °C, the samples were held at constant temperature for 9 h (*x* = 0), 3.5 h (*x* = 0.075), and 4 h (*x* = 0.25). The reduced time at 900 °C for *x* > 0 was in part chosen because the reactions neared completion and also to fit within the time allocated for the NPD experiment. Data collection continued during cooling down, which followed the natural cooling rate of the furnace. The temperatures quoted in the article are the average of start and end temperatures for each data set. The thermocouple was pressed against the V sample can. The samples experience uniform heating inside the vacuum furnace. No sudden increases in temperature were observed during the reaction, revealing the absence of any major exothermic events. After the experiments, the stack of pellets was found to have remained intact with the quarters somewhat fused together, showing some evidence of partial melting but not melted throughout. Rietveld refinement of the collected data (banks 4 and 5 of the GEM instrument were used) was done using GSAS software and the EXPGUI graphical user interface.^55,56^

### 2.2 Thermoelectric Properties

A separate set of TiNi_1+*x*_Sn (*x* = 0, 0.075, and 0.25) samples was prepared using the same powder methodology, followed by hot pressing at 900 °C and 80 MPa to obtain dense cylindrical disks (∼13 mm diameter × 1.5 mm thickness). The thermoelectric properties of these samples have been reported previously.^46^ Here, we reproduce this data and present further analysis building on the in situ NPD data analysis. Detailed structural characterization, including synchrotron X-ray powder diffraction (SXRD), NPD, and scanning electron microscopy, can be found in our earlier study.^46^ Of main relevance here is that these samples have identical lattice parameters and Ni interstitial concentrations to the samples reported here with hot pressing resulting in some broadening of the SXRD reflections but leaving the overall composition unchanged.

## 3 Results and Discussion

### 3.1 Neutron Powder Diffraction

**Figure 2:**
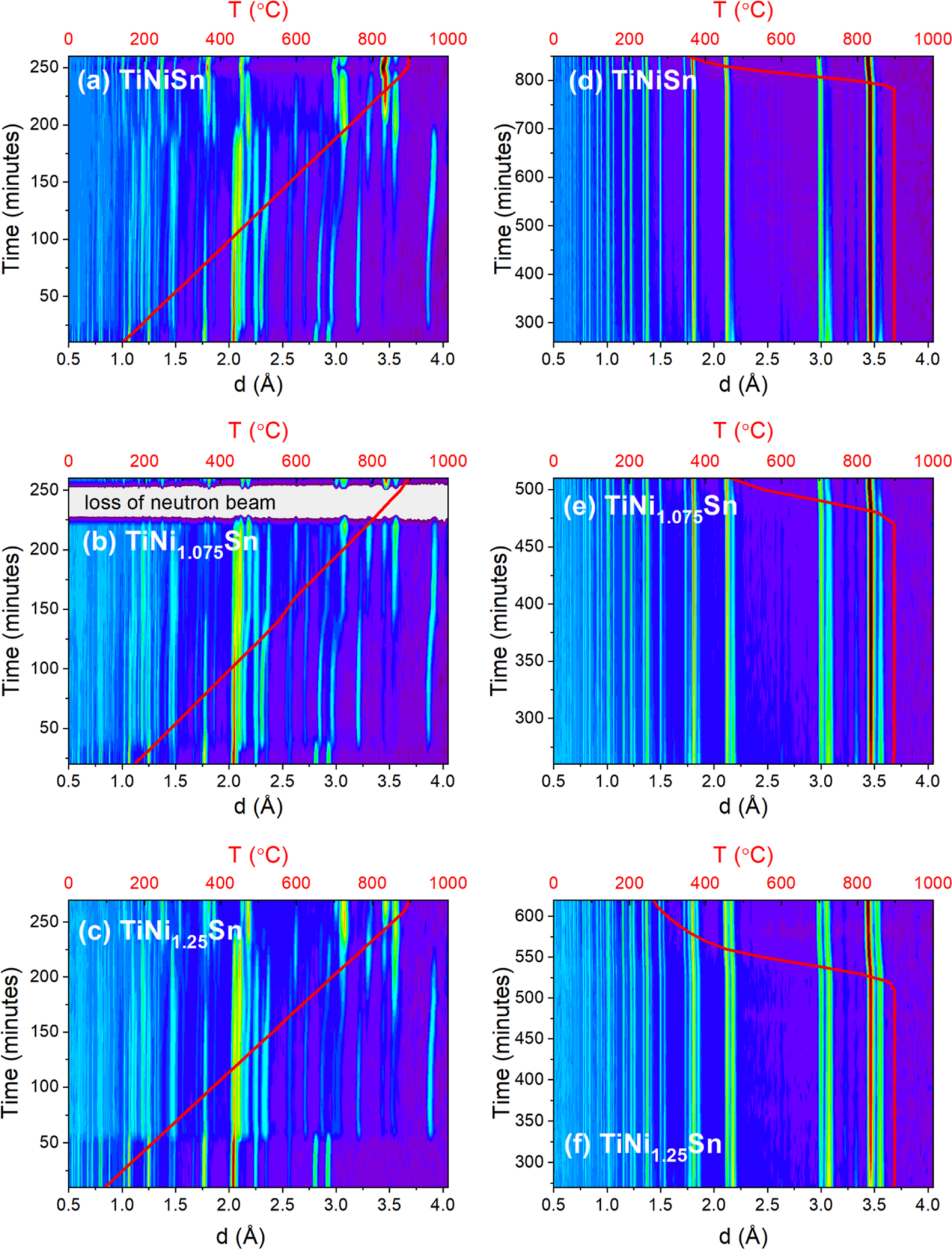
Intensity plots of the NPD data collected during synthesis of TiNi_1+*y*_Sn compositions from mixtures of elemental powder precursors. (a–c) show the changes during ramping at 3 °C/min to 900 °C. (d–f) show the evolution during heating at 900 °C and on cooling. Detailed phase information is presented in Figure 3.

**Figure 3:**
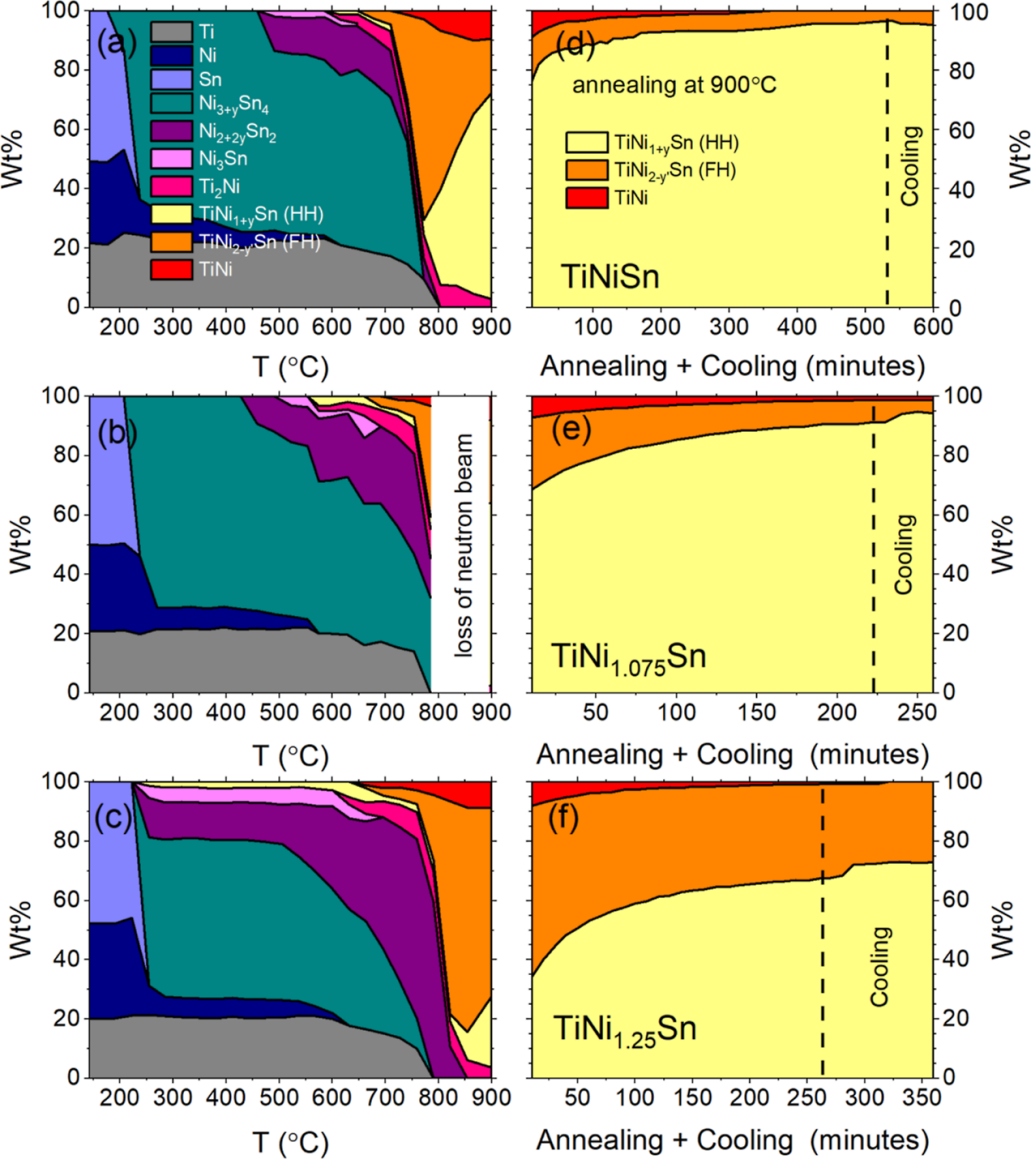
Phase evolution during synthesis of TiNi_1+*y*_Sn samples from mixtures of elemental powder precursors. (a–c) show the phase evolution during ramping at 3 °C/min to 900 °C. (d–f) show the gradual conversion to mixtures of HH and FH phases during heating at 900 °C and on cooling.

**Figure 4:**
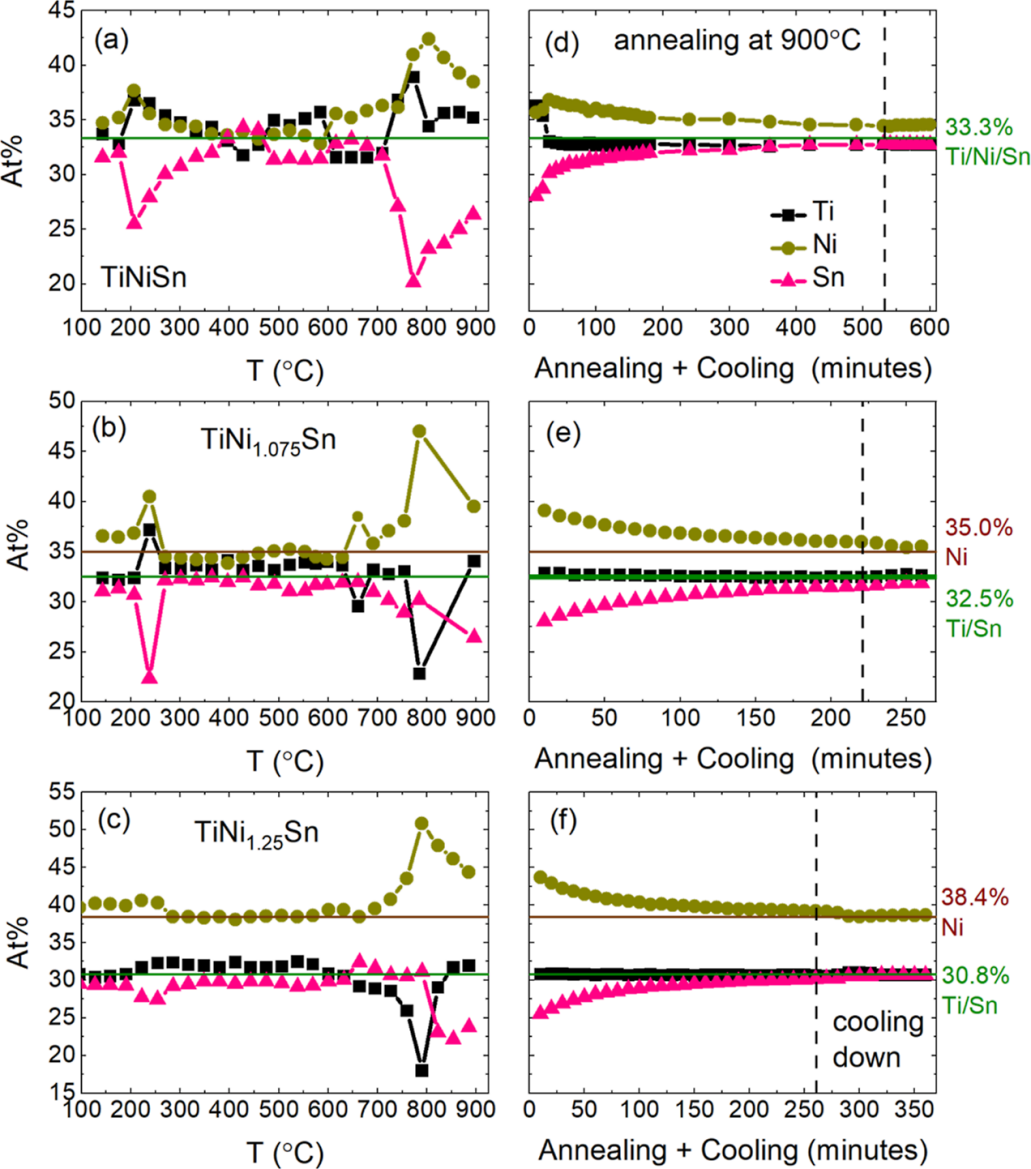
Temperature evolution of the atomic percentages of Ti, Ni, and Sn for the TiNi_1+*y*_Sn samples from Rietveld analysis of NPD data. (a–c) show the evolution during ramping at 3 °C/min to 900 °C. (d–f) show the evolution during heating at 900 °C and on cooling. Deviations from the nominal atomic percentages (indicated by horizontal lines) signal the presence of molten phases, not accounted for in the Rietveld phase analysis.

The diffraction data and quantitative phase evolution for the TiNi_1+*x*_Sn samples are summarized in Figures 2–4. In all cases, the left-hand-side panels are for data linked to ramping to 900 °C. The right-hand-side panels are for heating at 900 °C, followed by furnace cooling. The three rows are in order of increasing Ni content, i.e., *x* = 0, *x* = 0.075, and *x* = 0.25. Extended tables with fitted weight percentages (wt %), experimental compositions, and basic unit cell information are given in the Supporting Information (Tables S1–S7). Rietveld fits at 200, 600, 800 °C, and during annealing at 900 °C are given in Figures S1–S3 for all investigated compositions. Illustrations of the unit cells for all observed phases are shown in Figure S4. The temperature evolution of the lattice parameters of the phases observed during ramping and the Ni site occupancies for Ni_3_Sn_4_ and Ni_3_Sn_2_ are shown in Figures S5–S7.

The TiNi_1+*x*_Sn samples show similar phase evolution so are discussed together. The main focus is on TiNiSn with the specific influence of excess Ni pointed out where relevant. The temperature evolution of the diffraction patterns (detector bank 4 of the GEM instrument covering *d*-spacing between 0.4 and 6 Å) is shown as a color intensity plot in Figure 2. Visual inspection immediately reveals a number of sudden phase changes, in particular near the MP of Sn (232 °C) and then at 750–800 °C.

Figure 3 shows the phase evolution (wt %) obtained from Rietveld analysis, which confirms the two sudden transitions with gradual changes in between and above these temperatures. At 232 °C, Ni and Sn react to form Ni_3_Sn_4_, leaving Ti completely unreacted (Figure 3 and Table S1). This is consistent with the more refractive nature of Ti (MP = 1668 °C versus 1455 °C for Ni) but was unexpected due to the perceived high reactivity of Ti and the importance of Ti–Sn phases in the melt-based route. As the temperature is increased, Ni_3_Sn_4_ becomes enriched with Ni with up to 0.65 extra Ni incorporated per formula unit (Table S1 and Figure S5). As Ni_3_Sn_4_ absorbs more Ni and starts to approach a 1:1 Ni/Sn ratio, two further Ni–Sn binaries appear with Ni/Sn ratio >1. These are Ni_3_Sn_2_ and Ni_3_Sn (Figure 3 and Table S1 and Figure S5). The main impact of increasing the Ni content (*x*) on this first stage of the reaction is to increase the wt % of Ni_3_Sn_2_ and Ni_3_Sn, and lower the temperature at which they first appear, but globally, a very similar behavior is observed across all three samples. In all cases, Ni_3_Sn_4_ is the dominant binary phase (Figure 3; Tables S3 and S5). The first sign of the reaction of Ti occurs at ∼615 °C when small amounts (<5 wt % total) of Ti_2_Ni and HH TiNi_1+*y*_Sn appear, followed by ∼3 wt % FH TiNi_2*y*’_Sn at ∼680 °C (Table S1). At 750–800 °C, a sudden change occurs, with FH TiNi_2*y*’_Sn becoming the dominant phase, the Ni–Sn binaries completely disappearing, and TiNi emerging in addition to HH TiNi_1+*y*_Sn and Ti_2_Ni (Figure 3). This sudden change occurs near the MP for Ni_3_Sn_4_ (798 °C), consistent with the loss of Ni–Sn binaries from the diffraction patterns. Upon further heating to 900 °C, Ti_2_Ni disappears, and during annealing at 900 °C, TiNi and FH TiNi_2*y*’_Sn gradually convert into HH TiNi_1+*y*_Sn. In all three samples, TiNi fully disappears after ∼5 h annealing, with only minor changes in the HH/FH ratio beyond this point (Figure 3). This suggests that the reaction is completed after ∼5 h annealing at 900 °C.

Figure 4 shows the atomic percentages (at %) of Ti, Ni, and Sn throughout the heating, annealing, and cooling processes. These are based on the amount of Ti, Ni, and Sn contained in crystalline phases identified in the Rietveld fitting. This reveals an apparent loss of Sn and increase in the Ti and Ni content during the initial reaction stages at 230–400 °C, consistent with the presence of molten Sn (non-diffracting). This occurs in all three samples, but the effect is most evident for stoichiometric TiNiSn, whereas the higher-Ni content samples immediately fix the molten Sn in binary Ni–Sn compounds. The sudden change at 750–800 °C shows a similar pronounced loss of Sn for all samples. This again signals the presence of a molten component at this stage of the reaction, which is in keeping with the very fast emergence of TiNi, TiNi_2*y*’_Sn, and TiNi_1+*y*_Sn at this temperature. The Ti content behaves differently for the stoichiometric and Ni-rich samples. For *x* = 0, the Ti fraction increases, whereas for *x* > 0, a reduction is observed, signaling that some of the Ti is not incorporated in a diffracting phase. The main difference upon heating is the larger amount of Ni_3_Sn_2_ and Ni_3_Sn in the *x* > 0 samples. These binaries have a higher MP (Figure 1), perhaps slightly suppressing the initial supply of Ni in the reaction mixture when Ni_3_Sn_4_ melts, leading to formation of molten Ti–Sn instead of crystalline TiNi and Heusler phases. The presence of a molten Ti–Sn phase can also be inferred from the annealing stage, where TiNi and TiNi_2*y*’_Sn react to form TiNi_1+*y*_Sn. Mass balancing requires a source of Ti and Sn. Inspection of the phase diagram suggests that these may be Ti_2_Sn_3_ (MP = 751 °C) and Sn (MP = 232 °C), as all other Ti–Sn binaries have MP ≫ 900 °C.

To summarize, the main stages of the reaction are as follows:

1. *T* = 232 °C: reaction of Ni and Sn → Ni_3_Sn_4_ + Ni_3_Sn_2_ + Ni_3_Sn
2. *T* ∼ 600 °C: first reaction of Ti, emergence of Ti_2_Ni and small amounts of TiNi_1+y_Sn, followed by TiNi and TiNi_2y'_Sn at 700–750 °C
3. *T* ∼ 750–800 °C: sudden loss of Ni–Sn binaries to give three dominant phases: TiNi + TiNi_2*y*’_Sn + TiNi_1+*y*_Sn
4. *T* = 900 °C: conversion to the HH phase: TiNi + TiNi_2*y*’_Sn + 0.5Ti_2_Sn_3_ (l) + 0.5Sn (l) → TiNi_1+*y*_Sn

**Figure 5:**
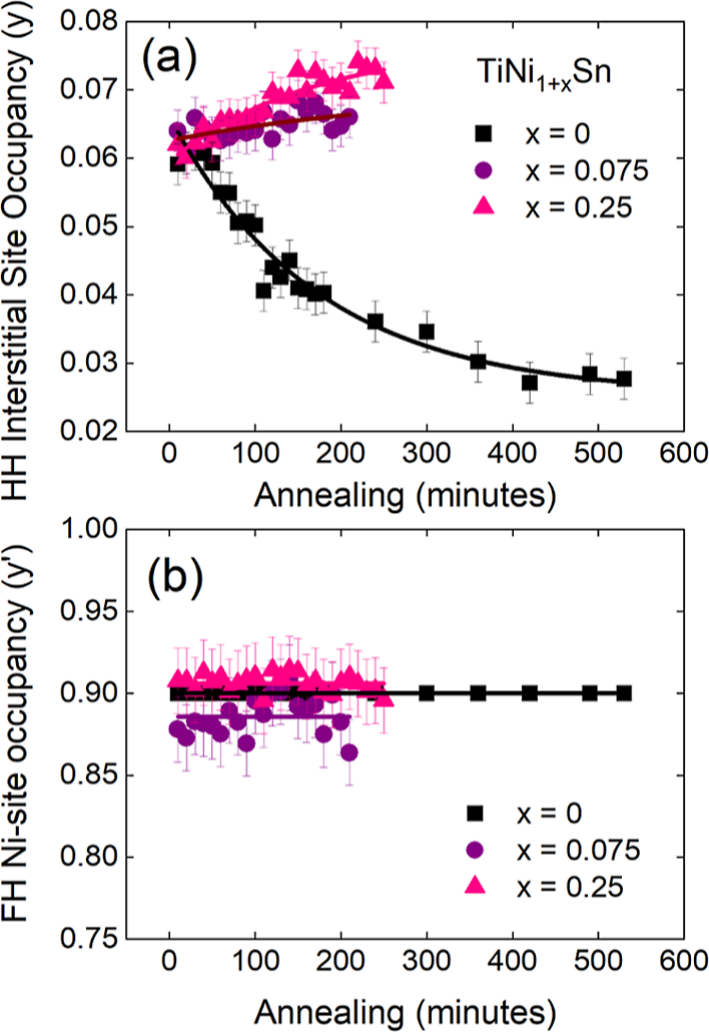
Evolution of the HH (TiNi_1+*y*_Sn) interstitial Ni content (*y*) and FH (TiNi_2*y*’_Sn) Ni-site occupancy (*y*’) for the TiNi_1+*x*_Sn samples during annealing at 900 °C. Lines in (a) are exponential fits. Lines in (b) are drawn at the average of the datapoints.

Monitoring of the reaction during the annealing and cooling stages provides important insights into the stability of Ni interstitials. Figure 5 shows the evolution of the Ni content in TiNi_1+*y*_Sn (interstitial Ni) and TiNi_2*y*’_Sn (Ni vacancies) during the annealing stage at 900 °C. Significantly, this reveals that the amount of interstitial Ni is identical upon initial HH phase formation (*y* ∼ 0.06); in other words, this does not depend on the nominal Ni content. Upon annealing, there are clear differences between the stoichiometric and Ni-rich samples. In the former, the Ni content reduces to an asymptotic value *y* ∼ 0.03, while in the other two samples, there is a gradual increase to *y* ∼ 0.07, reflecting the larger amount of Ni available in the reaction mixture (Figure 5a). The Ni-site occupancy of the FH structure, by contrast, is similar for the three samples with compositions close to TiNi_1.8_Sn during annealing (Figure 5b).

**Figure 6:**
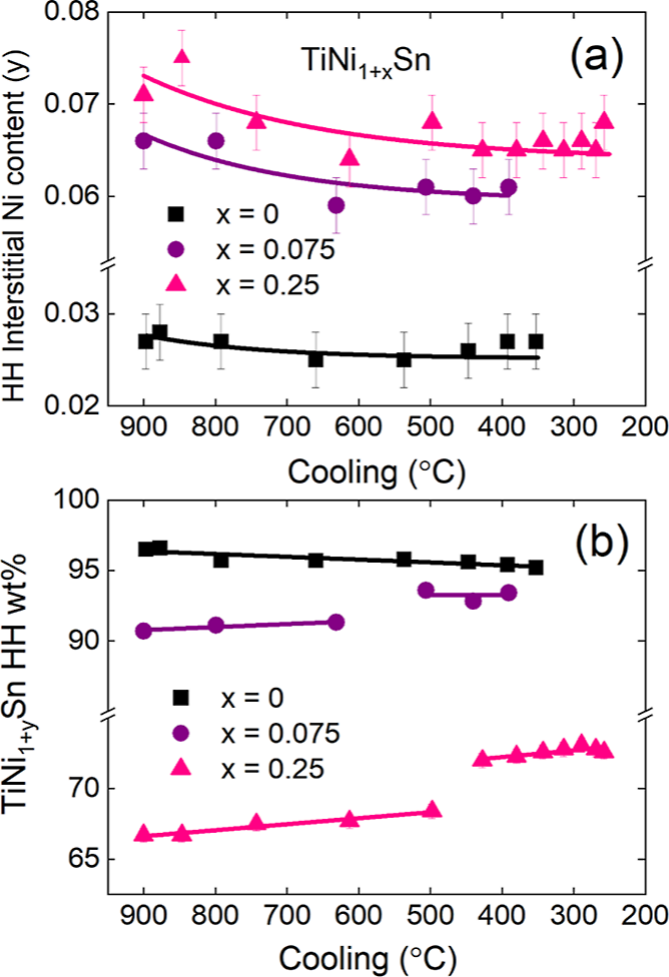
Evolution of the HH (TiNi_1+*y*_Sn) interstitial Ni content (*y*) and HH wt % for the TiNi_1+*x*_Sn samples during cooling after heating at 900 °C.

Figure 6 shows the interstitial Ni content and HH wt % on cooling from 900 °C. All samples show a small reduction in interstitial Ni on cooling, consistent with the behavior expected for an entropy-stabilized defect. Fitting using an exponential reveals a plateau below 500–600 °C, below which the Ni content is frozen (Figure 6a). Substantial and contrasting changes in HH wt % are also observed (Figure 6b). The *x* = 0 sample shows a small decrease of the amount of the HH phase on cooling (and corresponding increase in the FH phase), consistent with a reduced solubility of interstitial Ni and growth of FH domains. The Ni-rich samples show an unexpected increase in the HH fraction on cooling, interspersed by a step-change increase of 3–5 wt % at ∼600 °C (*x* = 0.075) and ∼500 °C (*x* = 0.25). This increase occurs at the expense of the FH phase, with no other phases evident in the diffraction patterns. This suggests that the HH phase has a greater stability at lower temperatures in the Ni-rich compositions. The cause of the discrete steps for *x* > 0 is not clear at present, but we have carefully checked our fits, and the effect appears real. We also note that *x* = 0, which was fitted in the same way does not have a corresponding jump, suggesting that the step for *x* > 0 is inherent to these compositions and not an artifact.

### 3.2 Thermoelectric Property Measurements

**Figure 7:**
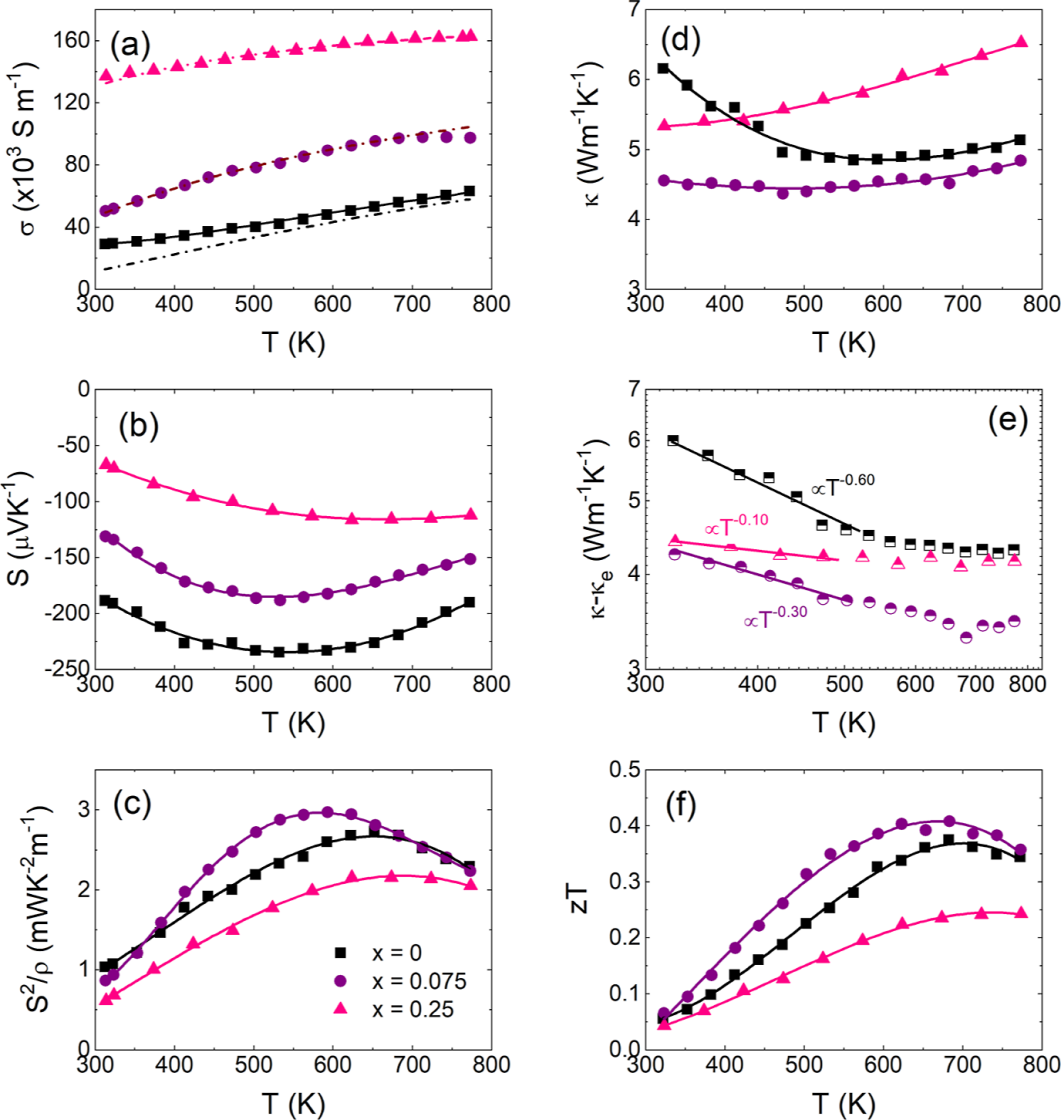
Measured thermoelectric properties of TiNi_1+*x*_Sn (*x* = 0, 0.075 and 0.25) samples. These were prepared using an analogous protocol to the in situ samples but with an additional hot-press treatment. (a) Electrical conductivity (σ), (b) absolute Seebeck coefficient (*S*), (c) thermoelectric power factor (*S*^*2*^σ), (d) thermal conductivity (κ), (e) subtraction of the electronic Lorenz component (κ*-*κ_e_), and (f) figure of merit (*zT*). The dashed–dotted lines in (a) indicate the intrinsic conductivity. The solid line for x = 0 is the total fitted conductivity. All other lines are guides to the eye.

Thermoelectric property data for TiNi_1+*x*_Sn (*x* = 0, 0.075, and 0.25) is presented in Figure 7. The impact of interstitial Ni has attracted a lot of attention in the HH literature.^38−42,46−48,51,57^ The current understanding is that it causes in-gap states leading to an impurity band, which reduces the effective bandgap to below the DFT value of 0.5 eV.^38,58,59^ It has also been established that the mobility of the p-type holes is about 5× as low as that of the n-type carriers.^59^ This is the key to the good performance of TiNiSn as it minimizes the degradation of *S*(*T*) and suppresses bipolar thermal conductivity (κ_bi_). The beneficial impact of a large electron/hole mobility ratio can be seen from the following expressions for σ, *S*, and κ_el_^60,61^
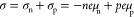
1
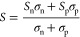
2
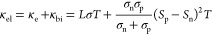
3Here, σ_n_ (σ_p_) and *S*_n_ (*S*_p_) are the electron (hole) electrical conductivity and Seebeck coefficient, respectively. σ_n_ (σ_p_) can be expressed in terms of the electron (hole) carrier concentration *n* (*p*) and mobility μ_n_ (μ_p_). The first term in eq 3 is the regular Lorenz contribution to κ_el_. In case of intrinsic semiconducting behavior, *n* = *p*, and a large difference in μ_n_ and μ_p_ mitigates against reductions in *S* and large κ_bi_.

Analysis of transport data has suggested that there is metal-like electronic conduction through the impurity band in addition to the regular valence and conduction bands.^58^ In order to accommodate these two conduction channels, σ(*T*) was fitted using a parallel degenerate (σ_deg_, metal-like) and semiconducting (σ_int_) channel^62^
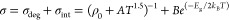
4Here, the *T*^1.5^ temperature dependence is typical for acoustic phonon scattering and ρ_0_ is the residual electrical resistivity at 0 K. The intrinsic term does not explicitly consider the high mobility ratio, but this minimal model provides a good fit to the data. The final fit parameters are given in Table 1, and the quality of the fit can be inspected in Figure 7a. In case of *x* = 0, ∼55% of the conduction at 300 K is through the impurity band, decreasing to <10% above 650 K. For *x* = 0.075 and *x* = 0.25, σ(*T*) is described adequately using σ_int_ without any contribution from the impurity band. This is consistent with the strongly reduced *E*_g_ = 0.14(1) eV (*x* = 0) to *E*_g_ = 0.07(1) eV (*x* = 0.075) and *E*_g_ = 0.02(1) eV (*x* = 0.25), yielding much larger concentrations of thermally excited carriers, hence drowning out any contribution of the impurity band. This is in keeping with Hall data on these samples,^46^ which show a decreasing Hall coefficient^61^
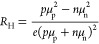
5The measured values decrease from *R*_H_ = 5.2 × 10^–2^ cm^3^ C^–1^ (*x* = 0) to *R*_H_ = 1.7 × 10^–2^ cm^3^ C^–1^ (*x* = 0.075) at 300 K. This signals an increase in the carrier concentration (from 1.2 × 10^20^ cm^–3^ to 3.7 × 10^20^ cm^–3^ in the single carrier limit), but it is difficult to disentangle the individual *n* and *p* contributions. An alternative way to obtain the bandgap is from *S*(*T*) using the Goldsmid–Sharp approach. However, this has to be corrected for the small-bandgap and large-electron–hole-weighted mobility ratios (*b* ∼ 5).^59^ Using the literature approach, values of 0.14(1) eV, 0.09(1) eV, and 0.03(1) eV are obtained; hence, the bandgap values from *S*(*T*) and our two-channel σ(*T*) fitting are in near perfect agreement. The *x* = 0.25 sample contains a large fraction of the metallic FH phase (∼25%). Nevertheless, the semiconducting σ(*T*) and *S*(*T*) and the bandgap follow the trend set by the two other samples very well, suggesting that the HH phase dominates the thermoelectric response. In future, it would be of interest to investigate higher *x*-values and analyze the properties using an effective medium model, e.g., as applied to the Ti_0.3_Zr_0.35_Hf_0.35_Ni_1+*x*_Sn composites.^63^ The overall conclusion is that intrinsic semiconducting transport is already significant near room temperature, and its impact rapidly increases with increasing amounts of interstitial Ni.

**Table 1:** Overview of Parameters Used to Fit the Electrical Conductivity of the TiNi_1+*x*_Sn Samples

	*x* = 0	*x* = 0.075	*x* = 0.25
ρ_0_ (mΩ m)	0		
*A* (× 10^–5^ mΩ m K^–1.5^)	1.1		
*B* (× 10^3^ S m^–1^)	160.4	174.2	187.9
*E*_g_ (eV) from σ(*T*)	0.14(1)	0.07(1)	0.02(1)
*E*_g_ (eV) from *S*(*T*)	0.14(1)	0.09(1)	0.03(1)

Signatures of the electronic transport can also be seen from κ(*T*), where all samples show an increase at high temperature (Figure 7d). For *x* = 0, a transition from decreasing to increasing κ(*T*) is found, while this is suppressed for the other samples, leading to an increasing κ(*T*) over the entire temperature range for *x* = 0.25. As indicated in eq 3, there are two electronic contributions to κ(*T*): κ_e_ can be readily calculated from *L*(*T*) and σ(*T*),^64^ whereas κ_bi_ relies on knowledge of *S*_p/n_(*T*) and σ_p/n_(*T*). Both electronic terms have a *∼T*^1^ temperature dependence, whereas κ_lat_ typically has a ∼*T*^–*n*^ dependence. Figure 7e shows κ(*T*) – κ_e_(*T*) = κ_lat_(*T*) + κ_bi_(*T*) on a log–log scale; hence, the slope gives the exponent (*n*). The fitted exponent near room temperature decreases from 0.6 (*x* = 0) to 0.3 (*x* = 0.075) to 0.1 (*x* = 0.25). A lower exponent is usually interpreted to result from increased structural disorder. For example, *n* = 1 for defect-free materials with Umklapp phonon scattering, and n = 0.5 for alloyed systems with point-defect disorder.^60^ The current values are therefore low, consistent with high degrees of structural disorder due to Ni interstitials and the mixed phase nature of *x* = 0.25. These low n-values border on a transition to glass-like behavior (*n* < 0). The unusual feature in κ(*T*)*-*κ_e_(*T*) is the absence of a strong κ_bi_ upturn for the *x* = 0.075 and 0.25 samples, which follow *T*^–*n*^ behavior up to 800 K. This suggests that κ_bi_ might be suppressed in the more disordered systems, even though these samples show a clear reduction in *S*(*T*). An alternative scenario is that κ_lat_(*T*) and κ_bi_(*T*) compensate, masking the bipolar increase. Quantitative analysis is difficult due to the lack of a clear transition between temperature domains dominated by κ_lat_ and κ_bi_. In addition, the most used κ_bi_ model is derived for a large bandgap (*E*_g_ > *k*_B_*T*),^65^ and it is not clear if this applies to the current set of materials. Further work, including measurements over a wider temperature range, is needed to fully disentangle the impact of κ_lat_(*T*) and κ_bi_(*T*) in this material system.

## 4 Discussion

We have investigated the formation of stoichiometric and Ni-rich TiNi_1+*x*_Sn samples. Phase formation follows a convoluted route with a critical role for molten phases. The behavior of interstitial Ni is controlled by thermodynamics and depends on nominal composition and temperature. We draw the following conclusions from our work:

- The melting of Sn at 232 °C and then of the Ni–Sn binaries at 750–800 °C enables rapid phase formation, by providing a solid–liquid reaction environment, which persists during the annealing stage at 900 °C, through the presence of Ti_2_Sn_3_ (l) and Sn (l). This enables fast reactions despite the presence of high-MP elements and compound phases. Final product formation is achieved within 3–5 h of reaching the annealing stage at 900 °C.

HH formation in the powder route differs from that in melt processing. The respective reactions in the annealing stage of the melt and powder routes are:

Melt: TiNi_2*y*’_Sn (s) + Ti_5_Sn_3_ (s) + Sn (l) → TiNi_1+*y*_Sn (s)
Powder: TiNi_2*y*’_Sn (s) + TiNi (s) + Ti_2_Sn_3_ (l) + Sn (l) → TiNi_1+*y*_Sn (s)

The main difference is the involvement of TiNi (MP = 1310 °C) and low-MP Ti_2_Sn_3_ phases in the powder route instead of the high-MP Ti/Sn > 1 binaries observed in melt processing. Neither route avoids the formation of an FH phase.

- A possible way to avoid FH TiNi_2_Sn is to react TiNi (s) and Sn (l) directly. This reaction has been attempted, and although direct formation of TiNiSn was observed at the TiNi/Sn interface, formation of the FH phase also occurred.^66,67^ This strongly suggests that there is no facile route to avoid FH formation in the Ti–Ni–Sn system, presumably on account of its greater stability at high temperature.
- ZrNiSn synthesized using self-propagating combustion has similar initial formation of Ni–Sn phases, but the subsequent steps are different with formation of ZrNiSn proposed to occur from balanced ratios of Zr, Sn, Ni_3_Sn_4_, and Ni_3_Sn_2_.^54^ The difference with TiNiSn could be because ZrNi is not reported as a stable phase, leaving all Zr available to react with the Ni–Sn phases at their MP. This is supported by the observed rapid formation of ZrNiSn, occurring on the scale of tens of minutes. Furthermore, FH formation is avoided as no Zr is trapped in a stable binary (as is the case for Ti in TiNi).
- The concentration of Ni interstitials at fixed temperature is controlled by nominal composition, in agreement with recent phase boundary mapping work (on arc-melted samples).^47^ This showed that stoichiometric TiNiSn forms with a few % interstitials, while slightly Ni- and Sn-deficient compositions (TiNi_0.98_Sn_0.99_) were found to be near stoichiometric. Nominal Ni-deficient TiNi_1-d_Sn has also been studied.^38,48^ In samples (*d* ≤ 0.08) consolidated in minutes, improved carrier mobilities suggesting a decrease of interstitial Ni were observed.^48^ Similar TiNi_1-d_Sn samples, but now annealed at 900 °C for extensive periods, also showed a reduction of Ni interstitials with stoichiometric TiNiSn observed for *d* = 0.3, but this was accompanied by the formation of substantial amounts of Ti_6_Sn_5_ impurity phases.^38^ Nevertheless, this does corroborate the conclusion that nominal stoichiometry dictates the final concentration of Ni interstitials in the HH structure. The excess Ni compositions studied here have an upper limit of 7% occupancy of the interstitial site (at 900 °C), in agreement with the phase boundary mapping study (6%)^47^ and earlier work (8%).^38,39,41,46,51^ The final interstitial Ni defect concentration and HH/FH amounts in the TiNi_1+*x*_Sn system are therefore under thermodynamic control [with composition (*x*) and temperature as control parameters] and do not depend on the used route (either arc melting or a direct reaction of powders). Stability calculations for TiNiSn predict a much lower solubility limit of 1–2% interstitial Ni at 900 °C,^68^ far below the 6–8% that is achievable. The agreement with experiments is much closer for ZrNiSn and HfNiSn, suggesting that cost of formation of Ni interstitials in TiNiSn is lower than calculated.
- The change in the HH/FH ratio on cooling shows that phase conversion is possible down to low temperatures of 500–600 °C. This must be driven by thermodynamics, as there is no compelling reason for increased kinetic effects on reducing temperature. This demonstrates that the HH and FH structures have different relative stability on cooling, depending on the nominal amount of Ni in the reaction mixture. In Ni-rich systems, the HH phase is more stable, while the reverse is true in the stoichiometric case.
- The presence of interstitial Ni strongly impacts on the observed thermoelectric properties. This includes evidence for an impurity band and strongly reduced *E*_g_ = 0.14–0.02 eV with consistent values from resistivity and Seebeck data. κ_lat_(*T*) becomes less temperature-dependent, suggesting that a glass-like κ_lat_ (∝ *T*^1^) might be possible if the concentration of interstitials can be increased, although this would likely be detrimental to the electronic properties. The absence of a strong increase due to bipolar effects, which would have been expected for such small-bandgap semiconductors, is also noteworthy.
- The ultimate thermoelectric performance of TiNiSn remains to be confirmed, but reported *zT* values do not lag far behind those of ZrNiSn or HfNiSn.^16,35,36^ In terms of the power factor,^48^ similar values have been reported in all three compositions, and it may be more of a challenge of processing optimization than that there are large fundamental differences in performance. This contrasts with TiCoSb, which is limited by a low intrinsic mobility and hence is unlikely to be ever as good as the heavier analogues, ZrCoSb and HfCoSb.^16^

To conclude, direct reaction between elemental powders is a viable route to produce TiNiSn-based half-Heusler alloys. Molten phases play a critical role at all stages of the reaction enabling fast phase formation, despite the presence of refractory metals and high-MP phases. Our results and the wider literature suggest that formation of TiNiSn always involves an FH phase and that it cannot be directly accessed, unlike ZrNiSn and HfNiSn. However, phase formation is under thermodynamic control at typical synthesis temperatures, allowing for control of the interstitial Ni content and systematic optimization of thermoelectric properties.

## Supporting Information Available

Crystallographic information from fits to NPD data; crystal structure drawings and unit cell information for all observed phases, representative Rietveld fits, and evolution of structural parameters of phases observed during the heating stage (PDF)

## Notes

Raw data on which this publication is based can be accessed via the Heriot-Watt University Data Repository (https://doi.org/10.17861/4a002c44-1870-4ff3-b588-7e54ed5d485d).

## References

[ref1] Su L. Z.; Wang D. Y.; Wang S. N.; Qin B. C.; Wang Y. P.; Qin Y. X.; Jin Y.; Chang C.; Zhao L. D. High thermoelectric performance realized through manipulating layered phonon-electron decoupling. Science 2022, 375, 1385–1389. 10.1126/science.abn8997. 35324303

[ref2] Jiang B. B.; Wang W.; Liu S. X.; Wang Y.; Wang C. F.; Chen Y. N.; Xie L.; Huang M. Y.; He J. Q. High figure-of-merit and power generation in high-entropy GeTe-based thermoelectrics. Science 2022, 377, 208–213. 10.1126/science.abq5815. 35857539

[ref3] Zhou C. J.; Lee Y. K.; Yu Y.; Byun S.; Luo Z. Z.; Lee H.; Ge B. Z.; Lee Y. L.; Chen X. Q.; Lee J. Y.; et al. Polycrystalline SnSe with a thermoelectric figure of merit greater than the single crystal. Nat. Mater. 2021, 20, 1378–1384. 10.1038/s41563-021-01064-6. 34341524 PMC8463294

[ref4] Roychowdhury S.; Ghosh T.; Arora R.; Samanta M.; Xie L.; Singh N. K.; Soni A.; He J. Q.; Waghmare U. V.; Biswas K. Enhanced atomic ordering leads to high thermoelectric performance in AgSbTe_2_. Science 2021, 371, 722–727. 10.1126/science.abb3517. 33574210

[ref5] Qin B. C.; Wang D. Y.; Liu X. X.; Qin Y. X.; Dong J. F.; Luo J. F.; Li J. W.; Liu W.; Tan G. J.; Tang X. F.; et al. Power generation and thermoelectric cooling enabled by momentum and energy multiband alignments. Science 2021, 373, 556–561. 10.1126/science.abi8668. 34326238

[ref6] Jiang B. B.; Yu Y.; Cui J.; Liu X. X.; Xie L.; Liao J. C.; Zhang Q. H.; Huang Y.; Ning S. C.; Jia B. H.; et al. High-entropy-stabilized chalcogenides with high thermoelectric performance. Science 2021, 371, 830–834. 10.1126/science.abe1292. 33602853

[ref7] Chang C.; Wu M. H.; He D. S.; Pei Y. L.; Wu C. F.; Wu X. F.; Yu H. L.; Zhu F. Y.; Wang K. D.; Chen Y.; et al. 3D charge and 2D phonon transports leading to high out-of-plane ZT in n-type SnSe crystals. Science 2018, 360, 778–783. 10.1126/science.aaq1479. 29773748

[ref8] Zhao L. D.; Tan G. J.; Hao S. Q.; He J. Q.; Pei Y. L.; Chi H.; Wang H.; Gong S. K.; Xu H. B.; Dravid V. P.; et al. Ultrahigh power factor and thermoelectric performance in hole-doped single-crystal SnSe. Science 2016, 351, 141–144. 10.1126/science.aad3749. 26612831

[ref9] Tang Y. L.; Gibbs Z. M.; Agapito L. A.; Li G.; Kim H. S.; Nardelli M. B.; Curtarolo S.; Snyder G. J. Convergence of multi-valley bands as the electronic origin of high thermoelectric performance in CoSb_3_ skutterudites. Nat. Mater. 2015, 14, 1223–1228. 10.1038/nmat4430. 26436339

[ref10] Kim S. I.; Lee K. H.; Mun H. A.; Kim H. S.; Hwang S. W.; Roh J. W.; Yang D. J.; Shin W. H.; Li X. S.; Lee Y. H.; et al. Dense dislocation arrays embedded in grain boundaries for high-performance bulk thermoelectrics. Science 2015, 348, 109–114. 10.1126/science.aaa4166. 25838382

[ref11] Liu H. L.; Shi X.; Xu F. F.; Zhang L. L.; Zhang W. Q.; Chen L. D.; Li Q.; Uher C.; Day T.; Snyder G. J. Copper ion liquid-like thermoelectrics. Nat. Mater. 2012, 11, 422–425. 10.1038/nmat3273. 22406814

[ref12] Biswas K.; He J. Q.; Blum I. D.; Wu C. I.; Hogan T. P.; Seidman D. N.; Dravid V. P.; Kanatzidis M. G. High-performance bulk thermoelectrics with all-scale hierarchical architectures. Nature 2012, 489, 414–418. 10.1038/nature11439. 22996556

[ref13] Pei Y. Z.; Shi X. Y.; LaLonde A.; Wang H.; Chen L. D.; Snyder G. J. Convergence of electronic bands for high performance bulk thermoelectrics. Nature 2011, 473, 66–69. 10.1038/nature09996. 21544143

[ref14] Funahashi R. Thermoelectric Energy Conversion; Woodhead Publishing, 2021; p 730.

[ref15] Rowe D. M. Materials, preparation, and characterization in thermoelectrics. Materials, Preparation, and Characterization in Thermoelectrics; CRC Press: Boca Raton, 2012; pp 1–553.

[ref16] Quinn R. J.; Bos J.-W. G. Advances in half-Heusler alloys for thermoelectric power generation. Mater. Adv. 2021, 2, 6246–6266. 10.1039/d1ma00707f.

[ref17] Zhu T.; Fu C.; Xie H.; Liu Y.; Zhao X. High Efficiency Half-Heusler Thermoelectric Materials for Energy Harvesting. Adv. Energy Mater. 2015, 5, 1500588 10.1002/aenm.201500588.

[ref18] Poon S. J. Half Heusler compounds: promising materials for mid-to-high temperature thermoelectric conversion. J. Phys. D Appl. Phys. 2019, 52, 493001 10.1088/1361-6463/ab3d71.

[ref19] Fu C. G.; Bai S. Q.; Liu Y. T.; Tang Y. S.; Chen L. D.; Zhao X. B.; Zhu T. J. Realizing high figure of merit in heavy-band p-type half-Heusler thermoelectric materials. Nat. Commun. 2015, 6, 8144 10.1038/ncomms9144. 26330371 PMC4569725

[ref20] Zhu H.; He R.; Mao J.; Zhu Q.; Li C.; Sun J.; Ren W.; Wang Y.; Liu Z.; Tang Z.; et al. Discovery of ZrCoBi based half Heuslers with high thermoelectric conversion efficiency. Nat. Commun. 2018, 9, 2497 10.1038/s41467-018-04958-3. 29950678 PMC6021448

[ref21] Zhu H. T.; Mao J.; Li Y. W.; Sun J. F.; Wang Y. M.; Zhu Q.; Li G. N.; Song Q. C.; Zhou J. W.; Fu Y. H.; et al. Discovery of TaFeSb-based half-Heuslers with high thermoelectric performance. Nat. Commun. 2019, 10, 270 10.1038/s41467-018-08223-5. 30655512 PMC6336844

[ref22] Mitra M.; Benton A.; Akhanda M. S.; Qi J.; Zebarjadi M.; Singh D. J.; Poon S. J. Conventional Half-Heusler alloys advance state-of-the-art thermoelectric properties. Mater. Today Phys. 2022, 28, 100900 10.1016/j.mtphys.2022.100900.

[ref23] Anand S.; Wood M.; Xia Y.; Wolverton C.; Snyder G. Double Half-Heuslers. Joule 2019, 3, 1226–1238. 10.1016/j.joule.2019.04.003.

[ref24] Chen K.; Zhang R.; Bos J.-W. G.; Reece M. J. Synthesis and thermoelectric properties of high-entropy half-Heusler MFe_1–*x*_Co_*x*_Sb (M = equimolar Ti, Zr, Hf, V, Nb, Ta). J. Alloys Compd. 2022, 892, 162045 10.1016/j.jallcom.2021.162045.

[ref25] Ferluccio D. A.; Halpin J. E.; Macintosh K. L.; Quinn R. J.; Don E.; Smith R. I.; Maclaren D. A.; Bos J. W. G. Low thermal conductivity and promising thermoelectric performance in A_*x*_CoSb (A = V, Nb or Ta) half-Heuslers with inherent vacancies. J. Mater. Chem. C 2019, 7, 6539–6547. 10.1039/c9tc00743a.

[ref26] Xia K.; Liu Y.; Anand S.; Snyder G. J.; Xin J.; Yu J.; Zhao X.; Zhu T. Enhanced Thermoelectric Performance in 18-Electron Nb_0.8_CoSb Half-Heusler Compound with Intrinsic Nb Vacancies. Adv. Funct. Mater. 2018, 28, 1705845 10.1002/adfm.201705845.

[ref27] Bos J. W. G.; Downie R. A. Half-Heusler thermoelectrics: a complex class of materials. J. Phys. Condens. Matter 2014, 26, 433201 10.1088/0953-8984/26/43/433201. 25273549

[ref28] Zhu T. J.; Liu Y. T.; Fu C. G.; Heremans J. P.; Snyder J. G.; Zhao X. B. Compromise and Synergy in High-Efficiency Thermoelectric Materials. Adv. Mater. 2017, 29, 1605884 10.1002/adma.201702816. 28783221

[ref29] Zheng Y.; Slade T. J.; Hu L.; Tan X. Y.; Luo Y.; Luo Z.-Z.; Xu J.; Yan Q.; Kanatzidis M. G. Defect engineering in thermoelectric materials: what have we learned?. Chem. Soc. Rev. 2021, 50, 9022–9054. 10.1039/d1cs00347j. 34137396

[ref30] Snyder G. J.; Toberer E. S. Complex thermoelectric materials. Nat. Mater. 2008, 7, 105–114. 10.1038/nmat2090. 18219332

[ref31] Snyder G. J.; Snyder A. H. Figure of merit ZT of a thermoelectric device defined from materials properties. Energy Environ. Sci. 2017, 10, 2280–2283. 10.1039/c7ee02007d.

[ref32] Yu C.; Zhu T.-J.; Shi R.-Z.; Zhang Y.; Zhao X.-B.; He J. High-performance half-Heusler thermoelectric materials Hf_1–*x*_Zr_*x*_NiSn_1–*y*_Sb_*y*_ prepared by levitation melting and spark plasma sintering. Acta Mater. 2009, 57, 2757–2764. 10.1016/j.actamat.2009.02.026.

[ref33] Schwall M.; Balke B. Phase separation as a key to a thermoelectric high efficiency. Phys. Chem. Chem. Phys. 2013, 15, 1868–1872. 10.1039/c2cp43946h. 23247074

[ref34] Chen L.; Gao S.; Zeng X.; Mehdizadeh Dehkordi A.; Tritt T. M.; Poon S. J. Uncovering high thermoelectric figure of merit in (Hf,Zr)NiSn half-Heusler alloys. Appl. Phys. Lett. 2015, 107, 041902 10.1063/1.4927661.

[ref35] Gürth M.; Rogl G.; Romaka V. V.; Grytsiv A.; Bauer E.; Rogl P. Thermoelectric high ZT half-Heusler alloys Ti_1–*x*–*y*_Zr_*x*_Hf_*y*_NiSn (0 ≤ x ≤ 1; 0 ≤ y ≤ 1). Acta Mater. 2016, 104, 210–222. 10.1016/j.actamat.2015.11.022.

[ref36] Rogl G.; Sauerschnig P.; Rykavets Z.; Romaka V. V.; Heinrich P.; Hinterleitner B.; Grytsiv A.; Bauer E.; Rogl P. (V,Nb)-doped half Heusler alloys based on {Ti,Zr,Hf}NiSn with high ZT. Acta Mater. 2017, 131, 336–348. 10.1016/j.actamat.2017.03.071.

[ref37] Kang H. B.; Poudel B.; Li W.; Lee H.; Saparamadu U.; Nozariasbmarz A.; Kang M. G.; Gupta A.; Heremans J. J.; Priya S. Decoupled phononic-electronic transport in multi-phase n-type half-Heusler nanocomposites enabling efficient high temperature power generation. Mater. Today 2020, 36, 63–72. 10.1016/j.mattod.2020.01.002.

[ref38] Downie R. A.; Smith R. I.; MacLaren D. A.; Bos J.-W. G. Metal Distributions, Efficient n-Type Doping, and Evidence for in-Gap States in TiNiM_*y*_Sn (M = Co, Ni, Cu) half-Heusler Nanocomposites. Chem. Mater. 2015, 27, 2449–2459. 10.1021/cm5045682.

[ref39] Birkel C. S.; Douglas J. E.; Lettiere B. R.; Seward G.; Verma N.; Zhang Y. C.; Pollock T. M.; Seshadri R.; Stucky G. D. Improving the thermoelectric properties of half-Heusler TiNiSn through inclusion of a second full-Heusler phase: microwave preparation and spark plasma sintering of TiNi1+xSn. Phys. Chem. Chem. Phys. 2013, 15, 6990–6997. 10.1039/c3cp50918d. 23552642

[ref40] Douglas J. E.; Birkel C. S.; Miao M. S.; Torbet C. J.; Stucky G. D.; Pollock T. M.; Seshadri R. Enhanced thermoelectric properties of bulk TiNiSn via formation of a TiNi_2_Sn second phase. Appl. Phys. Lett. 2012, 101, 183902 10.1063/1.4765358.

[ref41] Hazama H.; Matsubara M.; Asahi R.; Takeuchi T. Improvement of thermoelectric properties for half-Heusler TiNiSn by interstitial Ni defects. J. Appl. Phys. 2011, 110, 063710 10.1063/1.3633518.

[ref42] Xie H. H.; Wang H.; Fu C. G.; Liu Y. T.; Snyder G. J.; Zhao X. B.; Zhu T. J. The intrinsic disorder related alloy scattering in ZrNiSn half-Heusler thermoelectric materials. Sci. Rep. 2014, 4, 6888 10.1038/srep06888. 25363573 PMC4217114

[ref43] Downie R. A.; MacLaren D. A.; Smith R. I.; Bos J. W. G. Enhanced thermoelectric performance in TiNiSn-based half-Heuslers. Chem. Commun. 2013, 49, 4184–4186. 10.1039/c2cc37121a. 23287797

[ref44] Barczak S. A.; Halpin J. E.; Buckman J.; Decourt R.; Pollet M.; Smith R. I.; MacLaren D. A.; Bos J.-W. G. Grain-by-Grain Compositional Variations and Interstitial Metals—A New Route toward Achieving High Performance in Half-Heusler Thermoelectrics. ACS Appl. Mater. Interfaces 2018, 10, 4786–4793. 10.1021/acsami.7b14525. 29313341

[ref45] Barczak S. A.; Quinn R. J.; Halpin J. E.; Domosud K.; Smith R. I.; Baker A. R.; Don E.; Forbes I.; Refson K.; MacLaren D. A.; et al. Suppression of thermal conductivity without impeding electron mobility in n-type XNiSn half-Heusler thermoelectrics. J. Mater. Chem. A 2019, 7, 27124–27134. 10.1039/c9ta10128d.

[ref46] Barczak S. A.; Buckman J.; Smith R. I.; Baker A. R.; Don E.; Forbes I.; Bos J.-W. G. Impact of Interstitial Ni on the Thermoelectric Properties of the Half-Heusler TiNiSn. Materials 2018, 11, 536 10.3390/ma11040536. 29601547 PMC5951420

[ref47] Tang Y. L.; Li X. S.; Martin L. H. J.; Cuervo Reyes E.; Ivas T.; Leinenbach C.; Anand S.; Peters M.; Snyder G. J.; Battaglia C. Impact of Ni content on the thermoelectric properties of half-Heusler TiNiSn. Energy Environ. Sci. 2018, 11, 311–320. 10.1039/c7ee03062b.

[ref48] Ren W. Y.; Zhu H. T.; Mao J.; You L.; Song S. W.; Tong T.; Bao J. M.; Luo J.; Wang Z. M.; Ren Z. F. Manipulation of Ni Interstitials for Realizing Large Power Factor in TiNiSn-Based Materials. Adv. Electron. Mater. 2019, 5, 1900166 10.1002/aelm.201900166.

[ref49] Sauerschnig P.; Grytsiv A.; Vrestal J.; Romaka V. V.; Smetana B.; Giester G.; Bauer E.; Rogl P. On the constitution and thermodynamic modelling of the system Zr-Ni-Sn. J. Alloys Compd. 2018, 742, 1058–1082. 10.1016/j.jallcom.2017.12.012.

[ref50] Gurth M.; Grytsiv A.; Vrestal J.; Romaka V. V.; Giester G.; Bauer E.; Rogl P. On the constitution and thermodynamic modelling of the system Ti-Ni-Sn. RSC Adv. 2015, 5, 92270–92291. 10.1039/c5ra16074j.

[ref51] Douglas J. E.; Birkel C. S.; Verma N.; Miller V. M.; Miao M. S.; Stucky G. D.; Pollock T. M.; Seshadri R. Phase stability and property evolution of biphasic Ti-Ni-Sn alloys for use in thermoelectric applications. J. Appl. Phys. 2014, 115, 043720 10.1063/1.4862955.

[ref52] Downie R. A.; Barczak S. A.; Smith R. I.; Bos J. W. G. Compositions and thermoelectric properties of XNiSn (X = Ti, Zr, Hf) half-Heusler alloys. J. Mater. Chem. C 2015, 3, 10534–10542. 10.1039/c5tc02025e.

[ref53] Su X.; Fu F.; Yan Y.; Zheng G.; Liang T.; Zhang Q.; Cheng X.; Yang D.; Chi H.; Tang X.; et al. Self-propagating high-temperature synthesis for compound thermoelectrics and new criterion for combustion processing. Nat. Commun. 2014, 5, 4908 10.1038/ncomms5908. 25223333 PMC4175591

[ref54] Hu T.; Yang D.; Su X.; Yan Y.; You Y.; Liu W.; Uher C.; Tang X. Interpreting the Combustion Process for High-Performance ZrNiSn Thermoelectric Materials. ACS Appl. Mater. Interfaces 2018, 10, 864–872. 10.1021/acsami.7b15273. 29236464

[ref55] Larson A. C.; Von Dreele R. B. General Structure Analysis System (GSAS); Los Alamos National Laboratory Report LAUR 86-748, 2000.

[ref56] Toby B. H. EXPGUI, a graphical user interface for GSAS. J. Appl. Crystallogr. 2001, 34, 210–213. 10.1107/s0021889801002242.

[ref57] Berche A.; Jund P. Fully Ab-Initio Determination of the Thermoelectric Properties of Half-Heusler NiTiSn: Crucial Role of Interstitial Ni Defects. Materials 2018, 11, 868 10.3390/ma11060868. 29789503 PMC6025401

[ref58] Schrade M.; Berland K.; Kosinskiy A.; Heremans J. P.; Finstad T. G. Shallow impurity band in ZrNiSn. J. Appl. Phys. 2020, 127, 045103 10.1063/1.5112820.

[ref59] Schmitt J.; Gibbs Z. M.; Snyder G. J.; Felser C. Resolving the true band gap of ZrNiSn half-Heusler thermoelectric materials. Mater. Horiz. 2015, 2, 68–75. 10.1039/c4mh00142g.

[ref60] Tritt T. M. Thermal Conductivity: Theory, Properties, and Applications. Physics of Solids and Liquids; Springer: New York, NY, 2010; p 290.

[ref61] Ascroft N. W.; Mermin N. D. Solid State Physics; Holt, Rinehart and Winston, 1976.

[ref62] Quinn R. J.; Stenning G. B. G.; Bos J.-W. G. Electronic scattering in half-Heusler thermoelectrics from resistivity data. J. Phys.: Energy 2022, 4, 024005 10.1088/2515-7655/ac5f37.

[ref63] Appel O.; Zilber T.; Kalabukhov S.; Beeri O.; Gelbstein Y. Morphological effects on the thermoelectric properties of Ti_0.3_Zr_0.35_Hf_0.35_Ni_1+d_Sn alloys following phase separation. J. Mater. Chem. C 2015, 3, 11653–11659. 10.1039/c5tc03214h.

[ref64] Kim H. S.; Gibbs Z. M.; Tang Y. L.; Wang H.; Snyder G. J. Characterization of Lorenz number with Seebeck coefficient measurement. APL Mater. 2015, 3, 041506 10.1063/1.4908244.

[ref65] Glassbrenner C. J.; Slack G. A. Thermal Conductivity of Silicon and Germanium from 3K to the Melting Point. Phys. Rev. 1964, 134, A1058–A1069. 10.1103/physrev.134.a1058.

[ref66] Asami C.; Kimura Y.; Kita T.; Mishima Y. Diffusion Paths for the Formation of Half-Heusler Type Thermoelectric Compound TiNiSn. MRS Online Proc. Libr. 2008, 1128, 508 10.1557/proc-1128-u05-08.

[ref67] Kimura Y.; Asami C.; Chai Y. W.; Mishima Y. Thermoelectric Performance of Half-Heusler TiNiSn Alloys Fabricated by Solid-Liquid Reaction Sintering. Mater. Sci. Forum 2010, 654–656, 2795–2798. 10.4028/www.scientific.net/msf.654-656.2795.

[ref68] Page A.; Uher C.; Poudeu P. F.; Van Der Ven A. Phase separation of full-Heusler nanostructures in half-Heusler thermoelectrics and vibrational properties from first-principles calculations. Phys. Rev. B 2015, 92, 174102 10.1103/physrevb.92.174102.

[ref69] Bos J. W. G. In-situ Neutron Powder Diffraction Study of the Synthesis of Half-Heusler Thermoelectric Materials, STFC ISIS Neutron and Muon Source, 2016, 10.5286/ISIS.E.RB1610143.

