# Supplementary material for: Mechanistic Insights into the Formation of Thermoelectric TiNiSn from In Situ Neutron Powder Diffraction
Source: Chem Mater. 2023 Apr 26;35(9):3694–704. doi: 10.1021/acs.chemmater.3c00393 (PMC10173456; doi:10.1021/acs.chemmater.3c00393)
Supplement: Supplementary file 1 — cm3c00393_si_001.pdf [file cm3c00393_si_001.pdf]

Supporting Information for:

Mechanistic insight into the formation of thermoelectric TiNiSn from in-situ neutron powder diffraction

Sonia A. Barczak[1], Blair F. Kennedy[1], Ivan da Silva[2] and Jan-Willem G. Bos[1*]

[1] *Institute of Chemical Sciences and Centre for Advanced Energy Storage and Recovery, School of Engineering and Physical Sciences, Heriot-Watt University, Edinburgh, EH14 4AS, UK*

[2] *ISIS Facility, Rutherford Appleton Laboratory, Harwell Oxford, Didcot OX11 0QX, UK*

* Email: j.w.g.bos@hw.ac.uk

Table S1. *TiNiSn* –Weight percentage (wt%) of intermediate phases that were identified during the heating up of elemental precursors to form target TiNiSn alloy, determined through Rietveld analysis. T (°C) denotes the average temperature of 30°C measurement steps, y represents the Ni (2c) site occupancy of $Ni_{3+y}Sn_4$, Ni (2d) site occupancy of $Ni_{2+2y}Sn_2$, Ni (4d) site occupancy of $TiNi_{1+y}Sn$ and Ni (8d) site occupancy of $TiNi_{2y}Sn$. (f) denotes the atomic occupancy that was fixed (not allowed to refine).

T (ºC)	Ti	Ni	Sn	$Ni_{3+p}Sn_4$		$Ni_{2+2q}Sn_2$		Ni_3Sn	Ti_2Ni	$TiNi_{1+y}Sn$		$TiNi_{2y'}Sn$		TiNi
	wt%	wt%	wt%	wt%	p	wt%	q	wt%	wt%	wt%	y	wt%	y’	wt%
142.8	21.8	27.5	50.7											
175.5	21.2	27.8	51.1											
207.0	25.2	28.0	33.5	13.3	0.0 (f)									
237.6	24.4	12.0		63.5	0.0 (f)									
269.5	23.3	9.7		67.1	0.0 (f)									
301.0	22.7	8.8		68.5	0.040									
332.6	22.0	8.2		69.8	0.042									
364.2	22.2	7.3		70.5	0.054									
396.0	21.1	6.2		72.7	0.082									
427.3	20.0	5.5		74.4	0.126									
458.6	20.7	4.7		74.6	0.194									
490.2	22.7	3.3		60.6	0.230	11.5	0.579	1.9						
521.6	22.4	2.4		60.9	0.339	12.1	0.700	2.3						
552.9	22.8	1.7		60.7	0.417	12.3	0.626	2.4						
584.2	23.2	1.0		59.2	0.509	14.5	0.418	2.0						
615.6	21.0			57.2	0.567	16.9	0.368	1.7	1.9	1.2	0.0 (f)			
647.0	19.8			60.4	0.617	14.7	0.305	0.9	3.1	1.2	0.0 (f)			
678.5	18.4			57.3	0.617	15.3	0.311		4.7	1.7	0.0 (f)	2.6	0.9 (f)	
709.8	17.3			53.6	0.627	15.6	0.310		6.5	2.3	0.0 (f)	4.5	0.9 (f)	
741.2	14.7			41.0	0.592	3.8	0.337		7.3	3.6	0.0 (f)	28.3	0.9 (f)	1.3
772.5	9.7					6.9	0.50 (f)		7.8	5.1	0.0 (f)	67.7	0.9 (f)	2.8
803.6									7.6	32.1	0.048	53.5	0.9 (f)	6.8
834.7									7.3	46.1	0.056	38.2	0.9 (f)	8.3
866.0									4.6	60.6	0.058	24.8	0.9 (f)	10.0
894.0									2.9	69.7	0.062	18.1	0.9 (f)	9.4

Table S2. *TiNiSn* – Weight percentage (wt%) of intermediate phases that were identified during the annealing and cooling down of elemental precursors to form target TiNiSn alloy, determined through Rietveld analysis. T (°C) denotes the average temperature of 30°C measurement steps, while y represents Ni (4d) site occupancy of $TiNi_{1+y}Sn$ and Ni (8d) site occupancy of $TiNi_{2y}Sn$. (f) denotes the atomic occupancy that was fixed (not allowed to refine).

**Long annealing**					
**Time (minutes)**	**$TiNi_{1+y}Sn$**		**$TiNi_{2y'}Sn$**		**TiNi**
	**wt%**	**y**	**wt%**	**Y'**	**wt%**
10	76.3	0.059	14.8	0.9 (f)	8.9
20	82.2	0.061	10.7	0.9 (f)	7.1
30	84.3	0.061	9.8	0.9 (f)	5.9
40	85.8	0.061	9.1	0.9 (f)	5.1
50	86.5	0.059	9.3	0.9 (f)	4.3
60	87.4	0.055	8.9	0.9 (f)	3.7
70	87.0	0.055	9.3	0.9 (f)	3.6
80	87.6	0.050	9.0	0.9 (f)	3.4
90	88.9	0.051	7.9	0.9 (f)	3.2
100	88.6	0.050	8.6	0.9 (f)	2.9
110	89.6	0.041	7.9	0.9 (f)	2.5
120	89.1	0.044	8.8	0.9 (f)	2.3
130	90.7	0.043	7.0	0.9 (f)	2.2
140	90.5	0.045	7.6	0.9 (f)	1.9
150	91.0	0.041	6.9	0.9 (f)	2.1
160	91.2	0.041	6.7	0.9 (f)	2.0
170	92.6	0.040	5.7	0.9 (f)	1.7
180	92.8	0.040	5.6	0.9 (f)	1.6
240	93.4	0.036	5.5	0.9 (f)	1.1
300	93.1	0.035	6.1	0.9 (f)	0.9
360	94.0	0.035	6.0	0.9 (f)	
420	95.5	0.027	4.5	0.9 (f)	
490	95.8	0.028	4.1	0.9 (f)	
530	96.5	0.028	3.5	0.9 (f)	
**Cooling down**					
**T (°C)**	**$TiNi_{1+y}Sn$**		**$TiNi_{2y}Sn$**		**TiNi**
	**wt%**	**y**	**wt%**	**y**	**wt%**
897.0	96.5	0.027	3.5	0.9 (f)	
877.5	96.6	0.028	3.4	0.9 (f)	
792.1	95.7	0.027	4.3	0.9 (f)	
660.0	95.7	0.025	4.3	0.9 (f)	
537.0	59.8	0.025	4.2	0.9 (f)	
447.0	95.6	0.026	4.4	0.9 (f)	
392.5	95.4	0.027	4.6	0.9 (f)	
352.5	95.2	0.027	4.8	0.9 (f)	

Table S3. *$TiNi_{1.075}Sn$* – Weight percentage (wt%) of intermediate phases that were identified during the heating up of elemental precursors to form target $TiNi_{1.075}Sn$ alloy, determined through Rietveld analysis. T (°C) denotes the average temperature of 30°C measurement steps, y represents the Ni (2c) site occupancy of $Ni_{3+y}Sn_4$, Ni (2d) site occupancy of $Ni_{2+2y}Sn_2$, Ni (4d) site occupancy of $TiNi_{1+y}Sn$ and Ni (8d) site occupancy of $TiNi_{2y}Sn$. (f) denotes the atomic occupancy that was fixed (not allowed to refine). Data was not collected during heating stage in temperature range ~800 – 890 ºC.

T (ºC)	Ti	Ni	Sn	$Ni_{3+y}Sn_4$		$Ni_{2+2y}Sn_2$		Ni_3Sn	Ti_2Ni	$TiNi_{1+y}Sn$		$TiNi_{2y}Sn$		TiNi
	wt%	wt%	wt%	wt%	y	wt%	y	wt%	wt%	wt%	y	wt%	y	wt%
143.0	21.0	29.1	49.9											
175.0	20.8	28.9	50.3											
207.0	21.1	29.4	49.5											
238.1	19.8	26.1		54.0	0.136									
269.6	21.5	7.5		71.0	0.107									
301.6	21.4	7.3		71.2	0.103									
333.1	21.7	7.3		71.0	0.103									
363.9	21.3	7.2		71.5	0.109									
396.1	22.0	7.0		70.9	0.124									
427.6	21.3	7.0		71.7	0.145									
458.3	21.7	6.0		63.1	0.154	9.2	0.50 (f)							
491.0	21.4	5.1		61.6	0.224	11.9	0.50 (f)	3.1						
522.1	21.9	3.8		58.9	0.335	12.3	0.50 (f)	3.6						
553.3	22.0	2.8		58.4	0.412	13.1	0.50 (f)	2.5						
574.7	20.1			51.3	0.605	21.2	0.360	1.9	1.8	3.2	0.0 (f)			
597.7	20.1			51.6	0.605	21.4	0.352	1.4	2.0	3.0	0.0 (f)			
628.9	19.7			53.2	0.629	21.3	0.333	7.6	2.6	1.8	0.0 (f)			
660.1	16.1			47.6	0.653	22.2	0.321		3.6	2.8	0.0 (f)			
691.4	17.3			46.4	0.641	26.0	0.311		5.6	1.1	0.0 (f)	3.5	1.0 (f)	
722.7	15.5			40.8	0.638	30.0	0.304		7.2	1.9	0.0 (f)	3.4	1.0 (f)	1.1
754.1	13.9			32.9	0.662	33.9	0.294		9.0	3.5	0.0 (f)	5.2	1.0 (f)	1.6
784.7				32.2	0.646	13.2	0.250		9.8	4.0	0.0 (f)	37.5	1.0 (f)	3.2
816.1	Data were not collected													
847.5														
895.5									2.5	61.4	0.060	28.1	0.870	8.0

Table S4. *$TiNi_{1.075}Sn$* – Weight percentage (wt%) of intermediate phases that were identified during the annealing and cooling down of elemental precursors to form target $TiNi_{1.075}Sn$ alloy, determined through Rietveld analysis. T (°C) denotes the average temperature of 30°C measurement steps, while y represents Ni (4d) site occupancy of $TiNi_{1+y}Sn$ and Ni (8d) site occupancy of $TiNi_{2y}Sn$.

**Long annealing**					
**Time (minutes)**	**$TiNi_{1+y}Sn$**		**$TiNi_{2y}Sn$**		**TiNi**
	**wt%**	**y**	**wt%**	**y**	**wt%**
10	68.7	0.064	24.2	0.878	7.1
20	72.1	0.061	21.6	0.873	6.2
30	75.1	0.066	19.5	0.883	5.4
40	77.3	0.064	17.8	0.881	4.9
50	79.1	0.063	16.4	0.880	4.5
60	80.7	0.063	15.3	0.875	4.0
70	82.5	0.063	13.8	0.889	3.7
80	83.2	0.064	13.5	0.882	3.3
90	84.3	0.064	12.5	0.869	3.2
100	85.5	0.064	11.7	0.895	2.8
110	86.1	0.067	11.3	0.887	2.6
120	87.0	0.063	10.6	0.900	2.4
130	87.7	0.066	9.9	0.901	2.4
140	88.5	0.065	9.4	0.909	2.1
150	88.7	0.068	9.5	0.892	1.8
160	89.3	0.067	9.0	0.890	1.7
170	89.7	0.068	8.5	0.893	1.7
180	89.9	0.066	8.3	0.875	1.8
190	90.6	0.064	8.1	0.899	1.3
200	90.7	0.065	7.7	0.882	1.6
210	90.7	0.066	8.0	0.864	1.4
**Cooling down**					
**T (°C)**	**$TiNi_{1+y}Sn$**		**$TiNi_{2y}Sn$**		**TiNi**
	**wt%**	**y**	**wt%**	**y**	**wt%**
798.8	91.1	0.066	7.6	0.871	1.3
631.5	91.3	0.059	7.4	0.893	1.2
506.3	93.6	0.061	5.1	0.9 (f)	1.3
440.0	92.8	0.060	4.4	0.9 (f)	2.8
390.4	93.4	0.061	5.4	0.9 (f)	1.2

Table S5. *$TiNi_{1.25}Sn$* – Weight percentage (wt%) of intermediate phases that were identified during the heating up of elemental precursors to form target $TiNi_{1.25}Sn$ alloy, determined through Rietveld analysis. T (°C) denotes the average temperature of 30°C measurement steps, y represents the Ni (2c) site occupancy of $Ni_{3+y}Sn_4$, Ni (2d) site occupancy of $Ni_{2+2y}Sn_2$, Ni (4d) site occupancy of $TiNi_{1+y}Sn$ and Ni (8d) site occupancy of $TiNi_{2y}Sn$. (f) denotes the atomic occupancy that was fixed (not allowed to refine).

**T (ºC)**	**Ti**	**Ni**	**Sn**	**$Ni_{3+y}Sn_4$**		**$Ni_{2+2y}Sn_2$**		**Ni_3Sn**	**Ti_2Ni**	**$TiNi_{1+y}Sn$**		**$TiNi_{2y}Sn$**		**TiNi**
	**wt%**	**wt%**	**wt%**	**wt%**	**y**	**wt%**	**y**	**wt%**	**wt%**	**wt%**	**y**	**wt%**	**y**	**wt%**
95.7	20.1	31.8	48.0											
127.8	19.9	32.3	47.8											
158.4	20.0	32.2	47.7											
190.2	20.2	32.1	47.7											
222.3	21.1	33.1	45.8											
254.4	21.3	9.8		50.2	0.346	13.3	0.50 (f)	4.1		1.3	0.0 (f)			
285.2	20.9	6.5		53.1	0.377	12.6	0.50 (f)	5.0		1.9	0.0 (f)			
316.7	20.7	6.4		53.8	0.367	12.4	0.50 (f)	5.0		1.7	0.0 (f)			
348.3	20.4	6.5		54.0	0.366	12.2	0.459	5.0		1.9	0.0 (f)			
379.8	20.3	6.5		53.6	0.372	12.6	0.459	4.9		2.2	0.0 (f)			
411.7	20.8	6.4		53.2	0.375	12.8	0.476	4.8		2.0	0.0 (f)			
442.8	20.2	6.4		53.7	0.382	12.7	0.486	4.9		2.1	0.0 (f)			
474.6	20.3	6.2		53.2	0.387	13.2	0.440	5.2		1.9	0.0 (f)			
505.9	20.4	5.8		52.7	0.434	13.4	0.446	5.6		2.0	0.0 (f)			
537.5	21.1	4.9		48.8	0.487	17.8	0.386	5.7		1.7	0.0 (f)			
568.8	20.9	3.4		45.5	0.549	22.1	0.388	5.9		2.2	0.0 (f)			
600.3	20.0	2.0		41.9	0.606	27.8	0.374	5.5		2.8	0.0 (f)			
631.6	17.7			39.3	0.651	30.8	0.352	4.5	2.7	5.1	0.0 (f)			
663.0	16.5			36.4	0.654	33.8	0.341	2.6	3.7	4.9	0.0 (f)	1.0	1.0 (f)	1.0
695.1	15.2			28.5	0.690	44.4	0.322		5.4	2.0	0.0 (f)	2.4	1.0 (f)	2.1
726.5	13.6			19.2	0.707	52.1	0.306		7.5	1.9	0.0 (f)	3.9	1.0 (f)	1.8
759.4	10.0			10.1	0.759	60.6	0.303		9.0	2.8	0.0 (f)	4.7	1.0 (f)	2.7
790.2						59.6	0.309		10.1	4.0	0.0 (f)	21.9	0.954	4.4
822.1						10.8	0.262		8.5	2.4	0.0 (f)	71.9	0.952	6.4
853.6									6.1	9.4	0.0 (f)	76.0	0.928	8.5
884.8									3.6	24.2	0.063	63.3	0.901	8.9

Table S6. *$TiNi_{1.25}Sn$* – Weight percentage (wt%) of intermediate phases that were identified during the annealing and cooling down of elemental precursors to form target $TiNi_{1.25}Sn$ alloy, determined through Rietveld analysis. T (°C) denotes the average temperature of 30°C measurement steps, while y represents Ni (4d) site occupancy of $TiNi_{1+y}Sn$ and Ni (8d) site occupancy of $TiNi_{2y}Sn$.

**Long annealing**					
**Time (minutes)**	**$TiNi_{1+y}Sn$**		**$TiNi_{2y}Sn$**		**TiNi**
	**wt%**	**y**	**wt%**	**y**	**wt%**
10	34.4	0.062	57.6	0.908	8.0
20	40.1	0.060	53.0	0.908	6.9
30	44.5	0.062	49.6	0.902	5.9
40	48.4	0.065	46.5	0.913	5.1
50	50.7	0.062	44.7	0.907	4.6
60	53.1	0.065	43.1	0.910	3.8
70	54.6	0.066	41.7	0.901	3.6
80	56.5	0.065	40.2	0.906	3.4
90	57.7	0.066	39.6	0.909	2.7
100	59.0	0.066	38.2	0.911	2.7
110	59.7	0.067	37.9	0.895	2.4
120	61.2	0.070	36.7	0.914	2.1
130	61.7	0.069	36.3	0.910	2.0
140	62.7	0.069	35.3	0.915	2.0
150	63.3	0.073	35.1	0.913	1.6
160	63.8	0.070	34.6	0.904	1.6
170	64.5	0.073	34.0	0.908	1.5
180	64.6	0.071	34.0	0.901	1.4
190	65.1	0.070	33.5	0.899	1.3
200	65.5	0.072	33.4	0.907	1.1
210	66.0	0.070	32.9	0.910	1.1
220	66.2	0.074	32.8	0.906	0.9
230	66.6	0.073	32.4	0.901	1.0
240	66.8	0.073	32.2	0.902	0.9
250	66.7	0.071	32.4	0.896	0.9
**Cooling down**					
**T (°C)**	**$TiNi_{1+y}Sn$**		**$TiNi_{2y}Sn$**		**TiNi**
	**wt%**	**y**	**wt%**	**y**	**wt%**
846.9	66.7	0.075	32.4	0.904	0.9
742.7	67.5	0.068	31.9	0.913	0.7
612.7	67.7	0.064	31.7	0.910	0.5
497.6	68.4	0.068	30.9	0.903	0.7
427.5	72.0	0.065	27.2	0.909	0.7
379.7	72.3	0.065	27.0	0.917	0.7
343.0	72.6	0.066	26.7	0.931	0.7
314.0	72.8	0.065	27.2	0.945	
288.9	73.1	0.066	26.9	0.939	
269.0	72.8	0.065	27.2	0.935	
257.6	72.6	0.068	27.4	0.941	

Table S7. Space groups, lattice parameters and atomic sites from the ICSD database that formed the starting point for the Rietveld analysis of the variable temperature neutron powder diffraction data.

**Phase Name**	**Ti**					
**Space Group**	P63/mmc					
**Cell Parameters**	a (Å)	b (Å)	c (Å)	α (°)	β (°)	γ (°)
	2.951	2.951	4.682	90	90	120
**Elements**	X	Y	Z	Occupancy		
Ti (2c)	0.3333	0.6667	0.25	1.0		

**Phase Name**	**Ni**					
**Space Group**	Fm-3m					
**Cell Parameters**	a (Å)	b (Å)	c (Å)	α (°)	β (°)	γ (°)
	3.5238	3.5238	3.5238	90	90	90
**Elements**	X	Y	Z	Occupancy		
Ni (4a)	0.00	0.00	0.00	1.0		

**Phase Name**	**Sn**					
**Space Group**	I41/amd (Origin 2)					
**Cell Parameters**	a (Å)	b (Å)	c (Å)	α (°)	β (°)	γ (°)
	5.831	5.831	3.182	90	90	90
**Elements**	X	Y	Z	Occupancy		
Sn (4a)	0.00	0.75	0.125	1.0		

**Phase Name**	**$Ni_{3+y}Sn_4$**					
**Space Group**	C12/m1					
**Cell Parameters**	a (Å)	b (Å)	c (Å)	α (°)	β (°)	γ (°)
	12.199	4.0609	5.2238	90	105.170	90
**Elements**	X	Y	Z	Occupancy		
Ni (2a)	0.00	0.00	0.00	1.0		
Ni (2c)	0.00	0.00	0.50	y		
Ni (4i)	0.7855	0.00	0.1634	1.0		
Sn (4i)	0.4285	0.00	0.1864	1.0		
Sn (4i)	0.1718	0.00	0.3124	1.0		

**Phase Name**	**$Ni_{2+2y}Sn_2$ (Ni_3Sn_2)**					
**Space Group**	P63/mmc					
**Cell Parameters**	a (Å)	b (Å)	c (Å)	α (°)	β (°)	γ (°)
	4.146	4.146	5.253	90	90	120
**Elements**	X	Y	Z	Occupancy		
Ni (2a)	0.00	0.00	0.00	1.0		
Ni (2d)	0.3333	0.6667	0.75	0.5 or y		
Sn (2c)	0.3333	0.6667	0.25	1.0		

**Phase Name**	**Ni_3Sn**					
**Space Group**	P63/mmc					
**Cell Parameters**	a (Å)	b (Å)	c (Å)	α (°)	β (°)	γ (°)
	5.286	5.286	4.243	90	90	120
**Elements**	X	Y	Z	Occupancy		
Ni (6h)	0.1670	0.3340	0.25	1.0		
Sn (2c)	0.3333	0.6667	0.75	1.0		

**Phase Name**	**TiNi**					
**Space Group**	Pm-3m					
**Cell Parameters**	a (Å)	b (Å)	c (Å)	α (°)	β (°)	γ (°)
	3.01302	3.01302	3.01302	90	90	90
**Elements**	X	Y	Z	Occupancy		
Ti (1b)	0.50	0.50	0.50	1.0		
Ni (1a)	0.00	0.00	0.00	1.0		

**Phase Name**	**Ti_2Ni**					
**Space Group**	Fd-3m					
**Cell Parameters**	a (Å)	b (Å)	c (Å)	α (°)	β (°)	γ (°)
	11.307	11.307	11.307	90	90	90
**Elements**	X	Y	Z	Occupancy		
Ti (16c)	0.00	0.00	0.00	1.0		
Ni (32e)	0.213	0.213	0.213	1.0		
Ti (48f)	0.436	0.125	0.125	1.0		

**Phase Name**	**$TiNi_{1+y}Sn$**					
**Space Group**	F-43m					
**Cell Parameters**	a (Å)	b (Å)	c (Å)	α (°)	β (°)	γ (°)
	5.9309	5.9309	5.9309	90	90	90
**Elements**	X	Y	Z	Occupancy		
Ti (4a)	0.00	0.00	0.00	1.0		
Ni (4c)	0.25	0.25	0.25	1.0		
Ni (4d)	0.75	0.75	0.75	y		
Sn (4b)	0.25	0.50	0.50	1.0		

**Phase Name**	**$TiNi_{2y'}Sn$**					
**Space Group**	Fm-3m					
**Cell Parameters**	a (Å)	b (Å)	c (Å)	α (°)	β (°)	γ (°)
	6.08	6.08	6.08	90	90	90
**Elements**	X	Y	Z	Occupancy		
Ti (4a)	0.00	0.00	0.00	1.0		
Ni (8c)	0.25	0.25	0.25	y		
Sn (4b)	0.25	0.50	0.50	1.0		

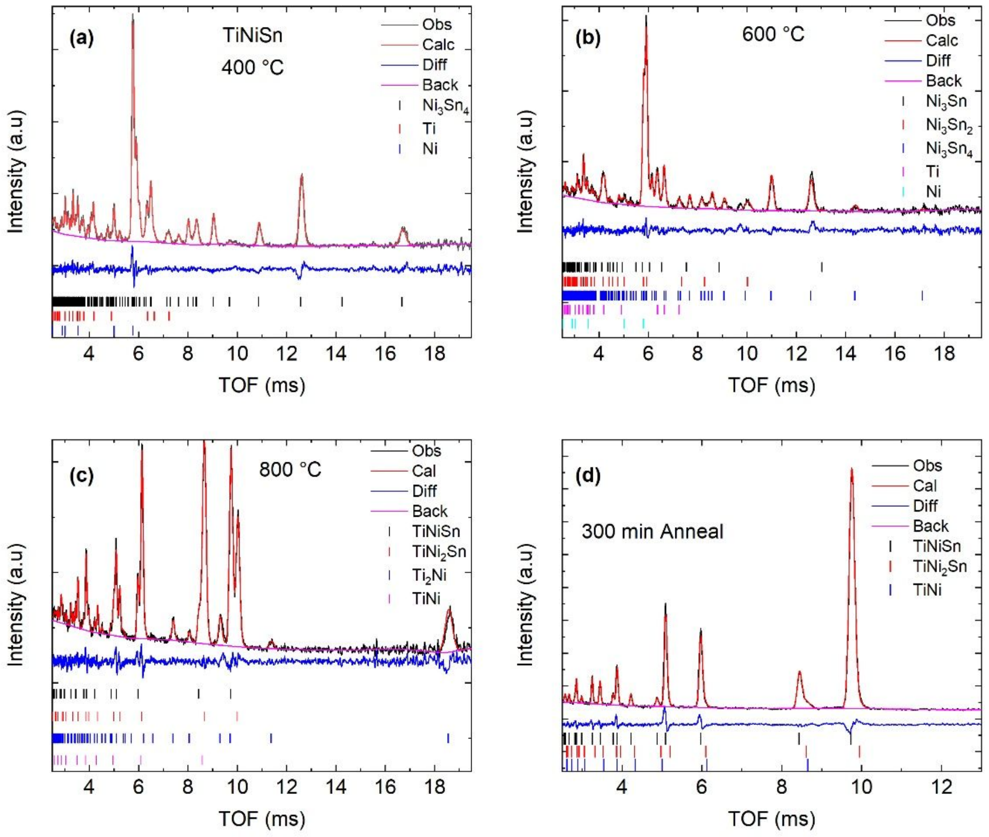

Fig. S1. Representative Rietveld fits to GEM neutron powder diffraction data collected on TiNiSn at 200, 600, 800 °C and after 5 hours annealing.

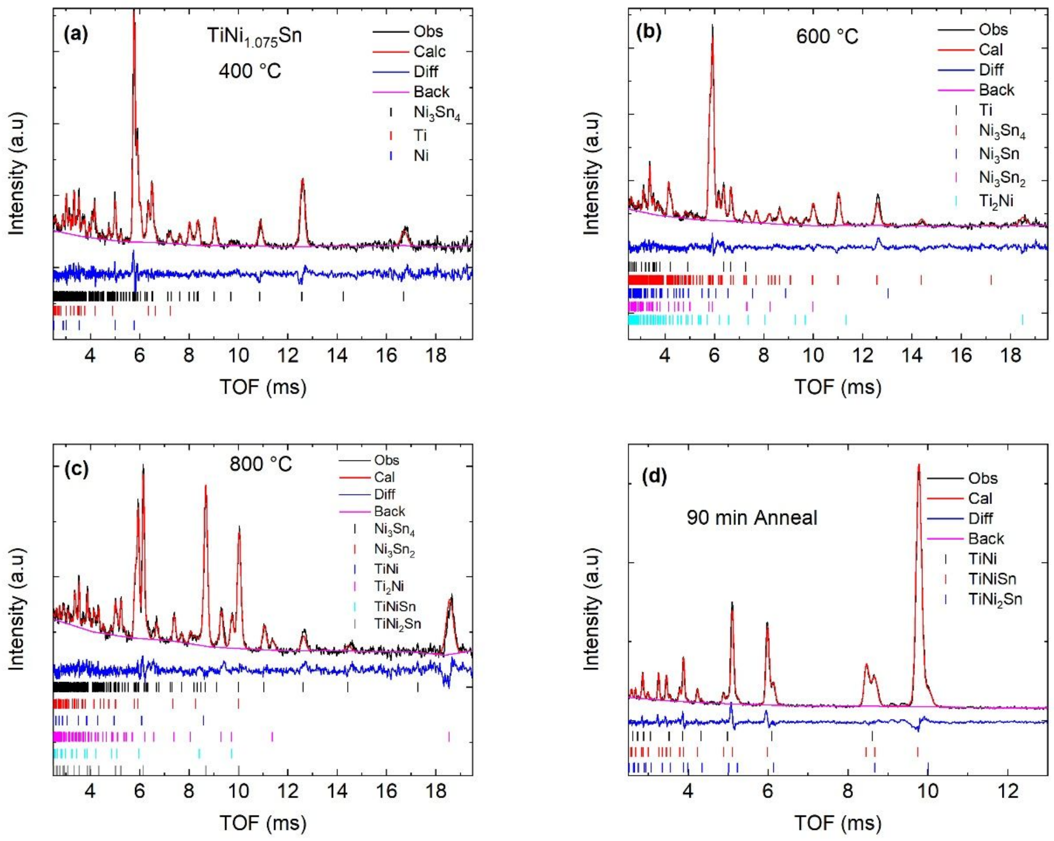

Fig. S2. Representative Rietveld fits to GEM neutron powder diffraction data collected on $TiNi_{1.075}Sn$ at 200, 600, 800 °C and after 1.5 hours annealing.

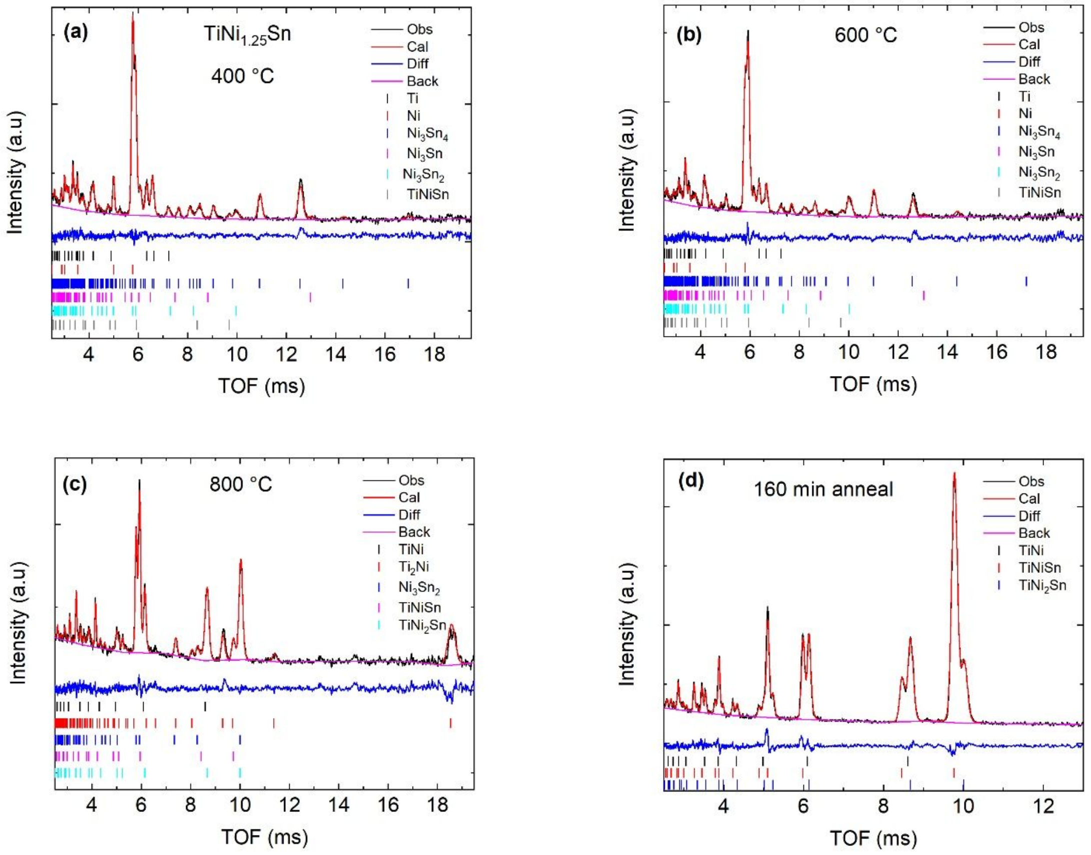

Fig. S3. Representative Rietveld fits to GEM neutron powder diffraction data collected on TiNi$_{1.25}$Sn at 200, 600, 800 °C and after 2 hr 40 mins annealing.

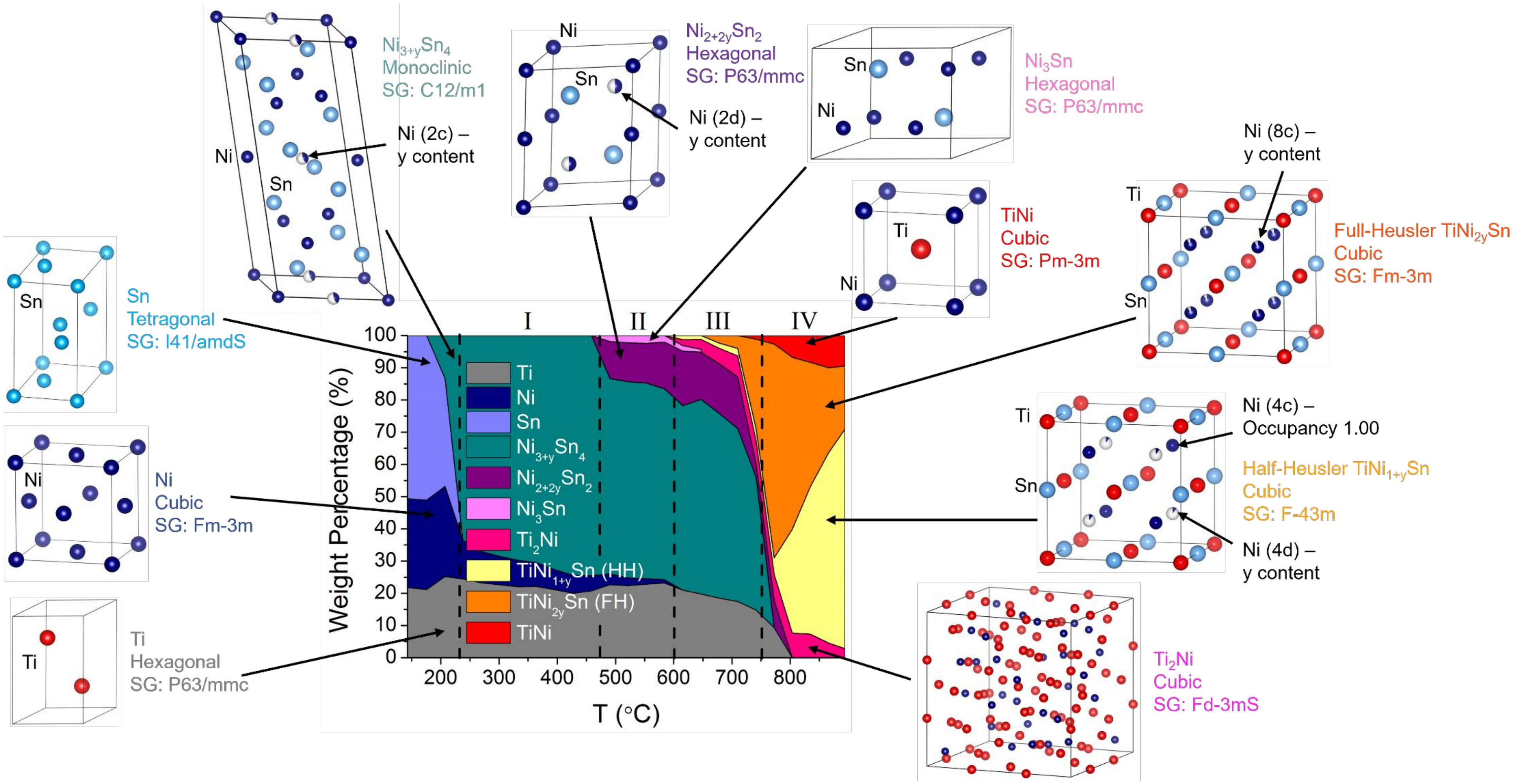

Fig. S4. Overview of the unit cells of the fitted phases used in this study.

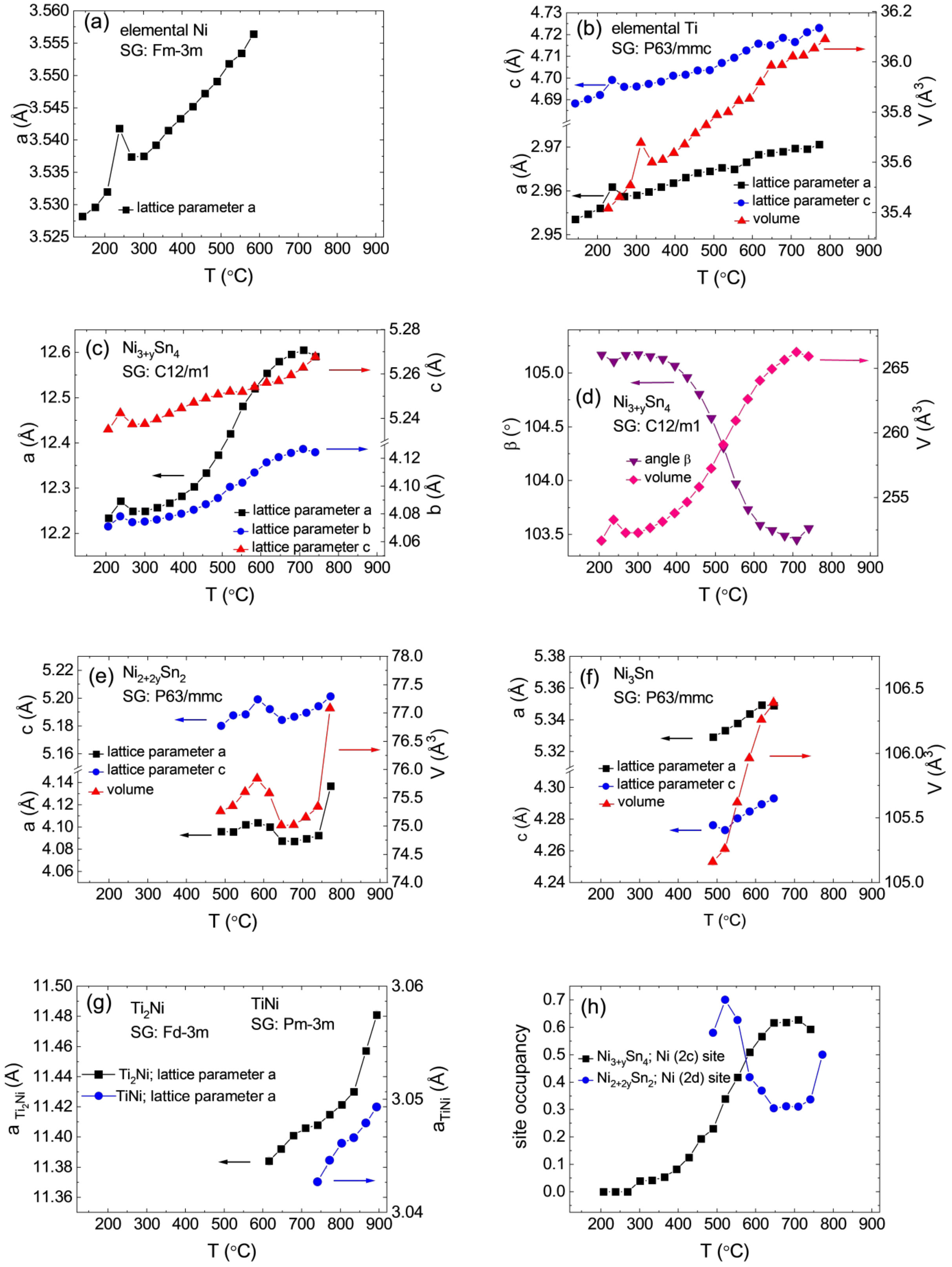

Fig. S5: *TiNiSn* – Temperature dependence of selected structural parameters of binary phases observed during ramping (3°C/min; 100°C - 900°C). Panels show lattice parameters for: **(a)** elemental Ni, **(b)** elemental Ti, **(c, d)** $Ni_{3+y}Sn_4$, **(e)** $Ni_{2+2y}Sn_2$, **(f)** Ni_3Sn and **(g)** Ti_2Ni and TiNi. Panel **(h)** shows the Ni (2c) site occupancy for $Ni_{3+y}Sn_4$ and Ni (2d) occupancy for $Ni_{2+2y}Sn_2$.

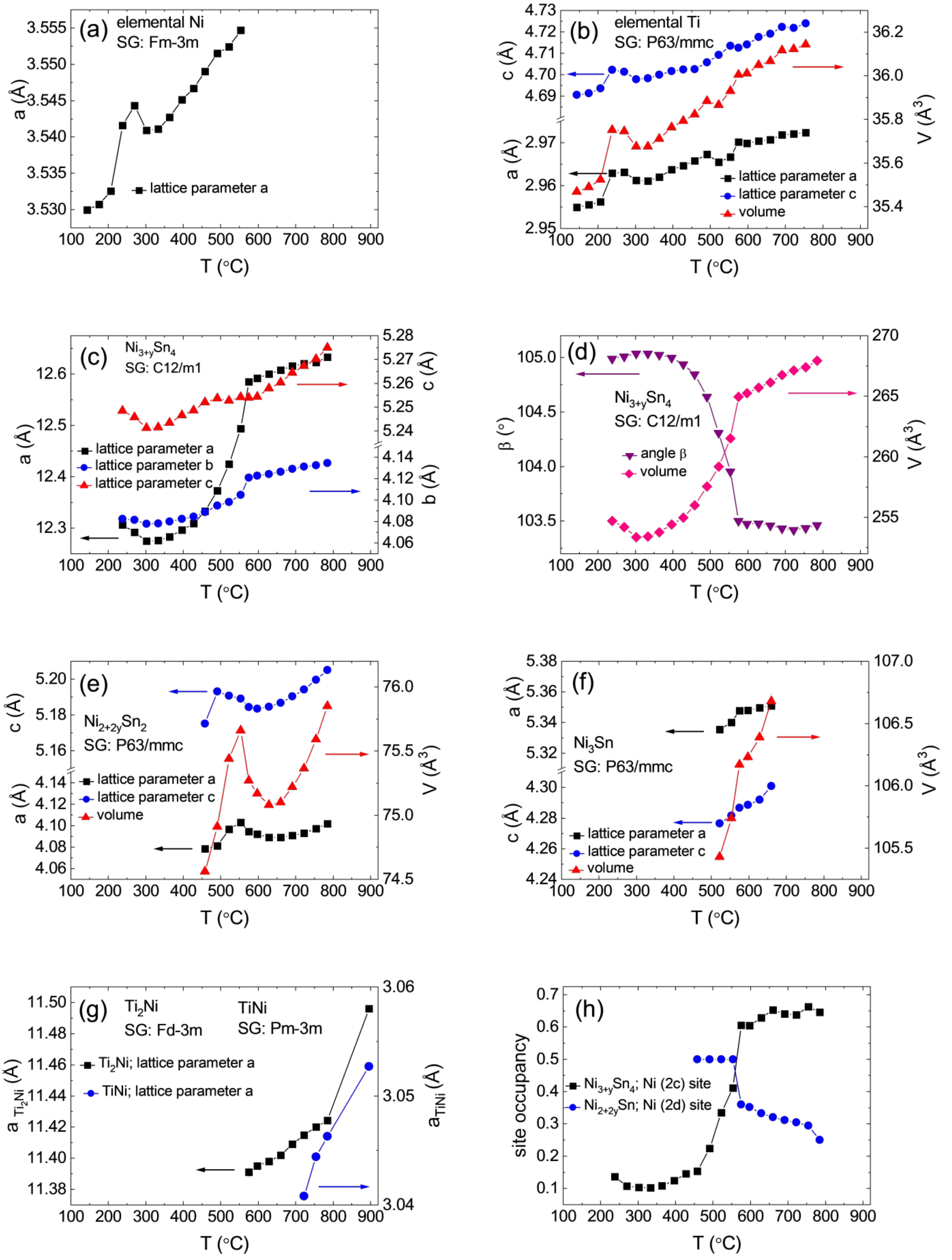

Fig. S6: *TiNi*$_{1.075}$*Sn* – Temperature dependence of selected structural parameters of binary phases observed during ramping (3°C/min; 100°C - 900°C). Panels show lattice parameters for: **(a)** elemental Ni, **(b)** elemental Ti, **(c, d)** $Ni_{3+y}Sn_4$, **(e)** $Ni_{2+2y}Sn_2$, **(f)** Ni_3Sn and **(g)** Ti_2Ni and TiNi. Panel **(h)** shows the Ni (2c) site occupancy for $Ni_{3+y}Sn_4$ and Ni (2d) occupancy for $Ni_{2+2y}Sn_2$.

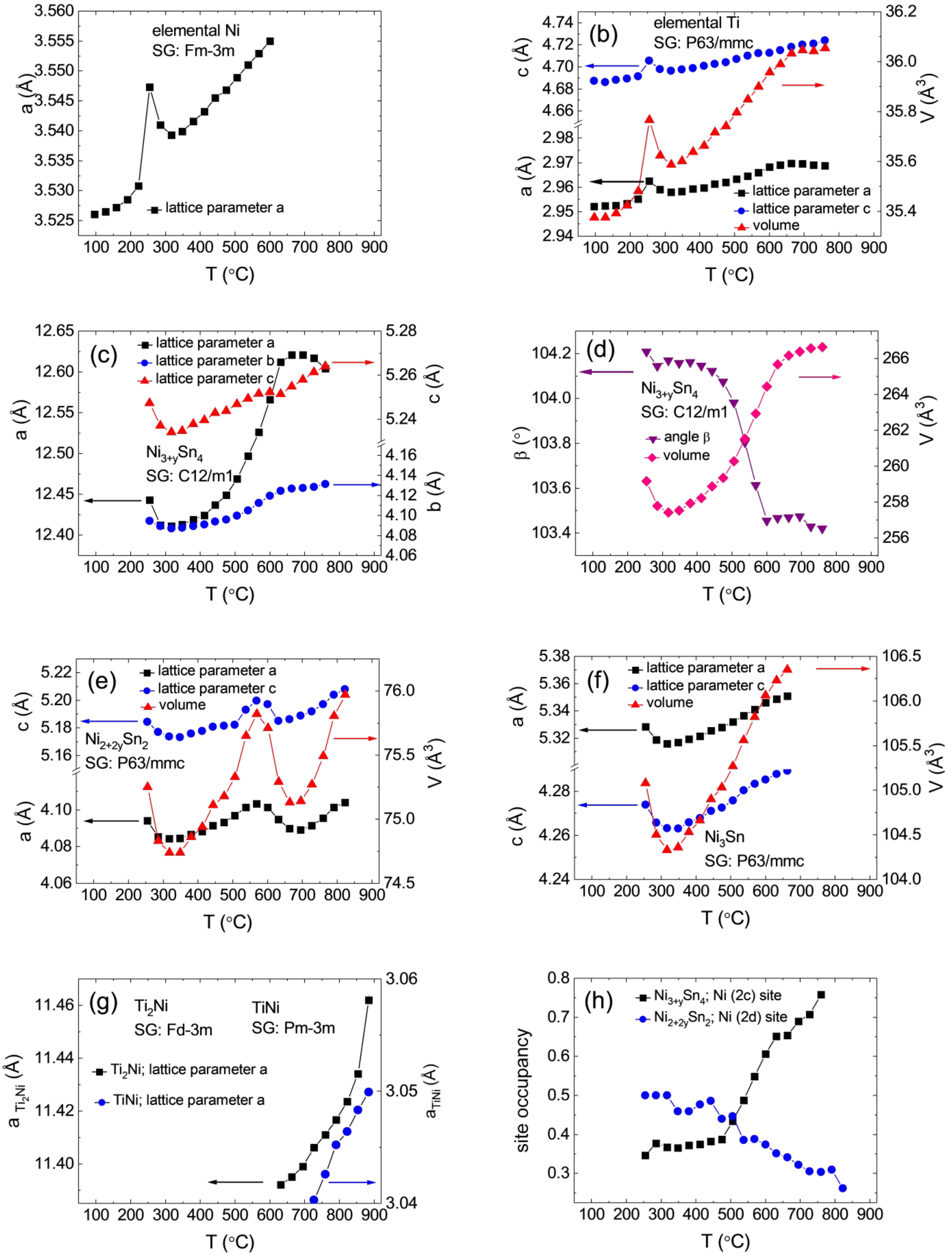

Fig. S7: *TiNi1.25Sn* – Temperature dependence of selected structural parameters of binary phases observed during ramping (3°C/min; 100°C - 900°C). Panels show lattice parameters for: **(a)** elemental Ni, **(b)** elemental Ti, **(c, d)** $Ni_{3+y}Sn_4$, **(e)** $Ni_{2+2y}Sn_2$, **(f)** Ni_3Sn and **(g)** Ti_2Ni and TiNi. Panel **(h)** shows the Ni (2c) site occupancy for $Ni_{3+y}Sn_4$ and Ni (2d) occupancy for $Ni_{2+2y}Sn_2$.
